# Do Initial Trunk Impairment, Age, Intervention Onset, and Training Volume Modulate the Effectiveness of Additional Trunk Exercise Programs after Stroke? A Systematic Review with Meta-Analyses

**DOI:** 10.3390/ijerph17238714

**Published:** 2020-11-24

**Authors:** Amaya Prat-Luri, Pedro Moreno-Navarro, Jose A. García, David Barbado, Francisco J. Vera-Garcia, Jose L.L. Elvira

**Affiliations:** 1 Department of Sport Sciences, Sports Research Centre, Miguel Hernández University of Elche, 03202 Elche, Spain; aprat@umh.es (A.P.-L.); p.moreno@umh.es (P.M.-N.); fvera@umh.es (F.J.V.-G.); jose.lopeze@umh.es (J.L.L.E.); 2 Infectious Diseases Unit, Hospital General Universitario de Elche, Miguel Hernández University of Elche, 03203 Elche, Spain; codicealberto@gmail.com

**Keywords:** core stability, rehabilitation, moderator

## Abstract

The aim of this systematic review is to analyze how, after additional trunk-focused training programs (ATEP), motor recovery after a stroke is modulated by potential effect modifiers. Twenty randomized controlled studies that carried out ATEP were included. Results showed moderate-to-high effects in favor of ATEP for trunk function, balance ability, gait performance, and functional mobility. Studies with a higher initial trunk impairment obtained a higher effect on trunk function and balance; studies with older participants had a higher effect on trunk function, limit of stability, and functional mobility, but not on balance ability. Older and more affected patients were, as well, those who started the intervention earlier, which was also linked with higher effects on trunk function, balance, and gait performance. Longer ATEP found a high effect on trunk function and balance ability. The potential effect modifiers seem to be important in the modulation of the effectiveness of ATEP and should be considered in the design of rehabilitation programs. Thus, since potential effect modifiers seem to modulate ATEP effectiveness, future studies should consider them in their experimental designs to better understand their impact on stroke rehabilitation.

## 1. Introduction

Commonly, trunk muscles are bilaterally affected after a stroke-onset, leading to an impairment of trunk function [1,2]. Since trunk structures are important to maintain the body in a stable state [3], the decreased trunk control experienced by this population (stroke patients) affects their ability to maintain balance [1,4]. The relevance of trunk control in this population is also supported by several longitudinal studies showing that the degree of trunk impairment seems to determine, to what extent, patients recover their motor function months after stroke-onset [5,6,7]. Based on these findings, the need to introduce trunk-focused exercises in stroke rehabilitation programs has increased [8,9,10,11].

Because of evidence leading towards an association between trunk function and motor performance in stroke patients, meta-analyses have been performed in order to obtain more in-depth knowledge about the effectiveness of additional trunk-focused exercise programs (ATEP) in conventional stroke rehabilitation programs to restore motor function [8,9,10,11]. In general, their results showed a positive impact of trunk-focused exercises on variables, such as trunk function, balance, and functional mobility. In this sense, a key point to optimize rehabilitation programs resides in the identification of potential effect modifiers, such as initial trunk impairment, age, and intervention onset or training volume that might modulate motor recovery [12]. However, little is known about which of these factors might induce a higher motor restoration after ATEP. To the authors’ knowledge, only Alhwoaimel et al., (2018) [9] performed sub-group analyses to observe the effects of the moment in which the rehabilitation program starts after stroke-onset, and the effects of total training volume on trunk function. Overall, they observed a higher effect on trunk function when rehabilitation started earlier, mainly when the program was applied in the acute phase after the stroke. Unexpectedly, they found that the shorter the rehabilitation programs were, the greater the recovery of the trunk function. In addition, it must be noted that these factors were only analyzed on trunk function, and the impact that they have on other relevant outcomes, such as balance or functional mobility, remains unknown [9]. Furthermore, although participants’ features, such as initial motor impairment or age, have been shown as relevant factors in the recovery process after a stroke, their potential relevance on modulating the effectiveness of ATEP has not been explored yet [12]. In this sense, analyzing how the initial trunk impairment (or the age at when people suffer the stroke) influence the improvement degree induced by ATEP could help to optimize this rehabilitation program, according to the stroke patients’ features [13]. Overall, although trunk exercises have shown to be effective in motor recovery, there is lack of evidence regarding what factors can influence ATEP in stroke rehabilitation.

Thus, the aim of this systematic review was to analyze the influence of potential effect modifiers as the initial trunk impairment, and participants’ age, the start of the intervention after stroke-onset, and the total volume of the ATEP on trunk function, balance ability, gait performance, and functional mobility in the stroke population. The analysis of these potential effect modifiers on ATEP could help in the optimization of stroke rehabilitation programs, maximizing their effectiveness.

## 2. Method

The current study was a systematic review carried out following the Preferred Reporting Items for Systematic Reviews and Meta-Analyses (PRISMA) guidelines (Table S1) [14].

### 2.1. Data sources and Searches

Different Boolean search strategies were employed for each of the databases (PubMed, Scopus, Cochrane Library, and EMBASE). Due to the broad amount of terms used to refer to the training of trunk structures (e.g., “core stability”, “core strength”, etc.) [15,16], a wide search combination was required as a strategy to avoid the loss of relevant articles (Table S2 of the Supplementary Materials). Furthermore, a manual search of the references was carried out to select any other potential study that could be included in the systematic review.

For the literature revision, two independent reviewers checked the titles and abstracts of the references to select any potentially relevant study. Afterwards, a full-text read was carried out on the selected documents. A third reviewer was consulted in case of disagreement.

### 2.2. Study Selection

The studies included had to meet the ensuing inclusion criteria: (1) the patients were in the stroke population; (2) they were peer-reviewed randomized controlled trials; (3) they included a control group receiving conventional rehabilitation; (4) they reported at least one outcome related to trunk function, balance, gait or functional mobility; (5) the experimental group performed a training program targeting the trunk structures as the main area, in addition to the conventional rehabilitation performed by the control group; (6) they had to be written in English, French, Italian, or Spanish. The following exclusion criteria were applied: (1) studies in which the experimental group targeted other body structures as the main area (e.g., upper or lower limbs); (2) studies analyzing a single session; (3) studies that did not provide pre- and post-intervention data. The search publication date was limited up to June 2020.

### 2.3. Data Extraction and Quality Assessment

The information extracted from the studies was registered in a codebook, including general data (e.g., the authors, year of publication, sample number, study-design, etc.), data from the intervention characteristics (e.g., number of training weeks, sessions per week, total training volume, type of exercises, and exercise progression), and pre- and post-intervention data (mean, standard deviation, and sample size) from the experimental and control groups.

The outcomes registered were trunk function, balance ability, gait performance, and functional mobility. To reduce within-outcome heterogeneity, when a study used more than one test/scale to analyze a specific outcome, the most frequent test/scale among the studies included was selected. Based on these criteria, the test/scales employed by the studies were:

- Trunk function was mainly assessed by the Trunk Impairment Scale and the Trunk Impairment Scale 2.0, which have been stated as valid and reliable tools to assess trunk motor impairment after a stroke [17,18]. The Trunk Control Test [19] was also employed.
- The Berg Balance Scale [20], the 3-level Berg Balance Scale [21], the Standing Equilibrium Index [22], and the Brief-BESTest [23] were employed for balance ability assessment. The limits of stability were evaluated through the Functional Reach Test [24], the modified Functional Reach Test [25], and the Lateral reach Test [26]. For limits of stability, three analyses were performed to observe the effects on the forward reach of the unaffected arm, and on the lateral reach of the unaffected arm and the affected arm respectively;
- Regarding gait performance, this outcome was assessed through the Functional Ambulation Categories test [27], the gait subscale of the Tinetti Scale [28], the 3-m walking test (m/s) [29], and the 10-m walking test (m/s) [30];
- Lastly, functional mobility was assessed with the Timed Up and Go Test (TUG) [31].

All assessment methods, with exception of the TUG and gait performance, implied a better motor function if the score was higher, but all were expressed in positive values if there was an improvement, and in negative values if there was a deterioration.

### 2.4. Risk of Bias and Quality of Evidence

The methodological quality of the studies was assessed with the Physiotherapy Evidence Database (PEDro) scale to evaluate the risk of bias, which establishes a maximum score of 10 points. Based on this scale, studies were categorized as follows: excellent (9–10 points), good (6–8 points), fair (4–5 points), and poor (<4 points) [32].

The quality of the evidence for each outcome was analyzed through the Grading of Recommendations, Assessment, Development, and Evaluation (GRADE) approach [33]. A.P. and P.M. analyzed and ranked the quality of evidence as very low, low, moderate, or high based on the score of the five GRADE items addressed: (1) risk of bias (PEDro scale), (2) inconsistency, (3) indirectness, (4) imprecision, (5) publication bias.

### 2.5. Data Synthesis and Analysis

The mean change and the standard deviation (SD) of the changes were taken from each study. Nonetheless, when this information was not provided, data from the pre- and post-test of the experimental and control groups were used to calculate the mean change and the standard deviation (SD) of the changes of each group. In these cases, as proposed by Rosenthal (1991) [34], an R-value equal to 0.7 was employed to estimate the SD of the changes. A correction factor was applied (c(gl) = 1 − (3/(4×(n − 1) − 1)) to avoid the bias of an overestimated pooled effect size [35].

The Review Manager (RevMan) software, (version 5.3, the Nordic Cochrane Centre, the Cochrane Collaboration, Copenhagen, Denmark, 2014), was used for the meta-analyses. The mean and SD of the changes for both the experimental and the control groups of each study were used to obtain the pooled effect sizes and their confidence intervals at 95%. A random-effect model was used in all cases because of the heterogeneity in the interventions and the inter-studies sample heterogeneity. The pooled effect size of each outcome was calculated using the weighted mean difference (MD) or the weighted standardized mean differences (SMD), depending on if the studies employed the same or different test, respectively. The standardized pooled effect sizes were categorized as follows: trivial: <0.20, low effect: 0.20–0.50, medium effect: 0.51–0.80, and high effect: >0.80 [36]. Finally, between-studies, heterogeneity was checked via the I^2^ statistic, categorized as none, low, moderate, and high for 0%, 25%, 50%, and 75%, respectively [37]. Additionally, in order to provide more clinically meaningful information that can be used by healthcare professionals, the pooled effect size was also calculated as the percentage of the pre-post change relative to each maximum scale score as showed by the intervention group compared to the control group. This index was calculated for those outcomes that used scales, which were trunk control and balance ability.

The potential effect modifiers (i.e., initial trunk impairment, participants’ age, the start of the intervention after stroke-onset, and total volume of training of additional trunk exercises) were analyzed through subgroup analyses. The initial trunk impairment was calculated as the average pre-test score of the trunk function outcome and transformed to relative score (%) in order to homogenize all the scales employed. Participants’ age was obtained from descriptive data. The start of the intervention after stroke-onset was obtained from the descriptive data and, when it was necessary, transformed into days. Lastly, the total volume of ATEP was calculated from the session duration, the frequency of the training and the duration (in weeks) of the program. All potential effect modifiers data, except the total volume of training (which only included the total ATEP duration), were averaged from the experimental and control groups. Afterwards, two groups were created based on the median score obtained from the data of all the studies (group A: articles below the median; group B: articles over the median).

## 3. Results

**Figure 1:**
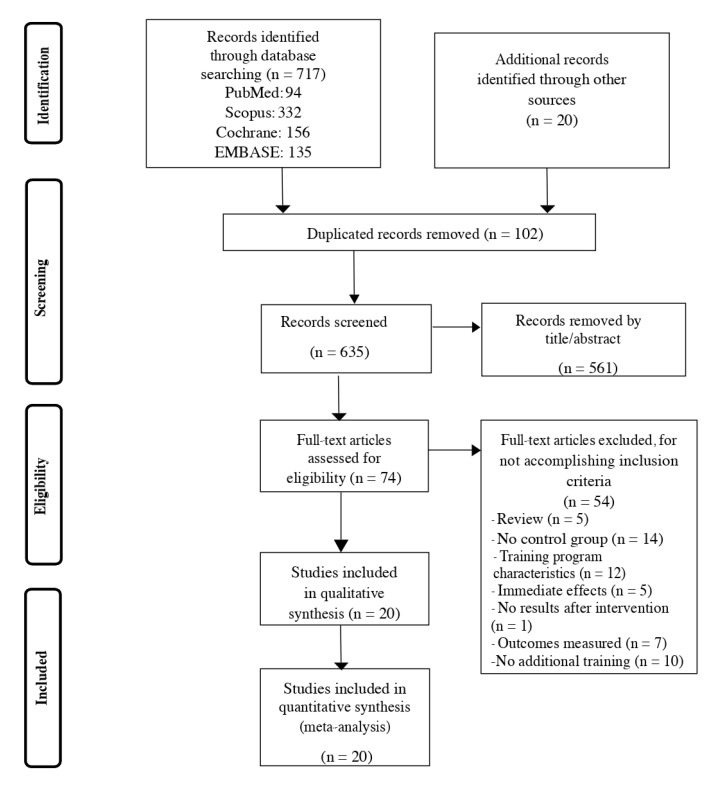
Flow chart diagram.

A total of 737 studies were initially identified (Figure 1) through both database searching (n = 717) and additional search of studies (n = 20), out of which 102 were duplicated records. From the 635-screened studies, 561 studies were removed after title/abstract reading. After a more detailed reading, 54 of them were excluded for different reasons (5 were systematic reviews; 14 did not have a control group; 12 did not match the characteristics of the training program (e.g., they included cardiovascular exercises, lower or upper limb strengthening, etc.); 5 only evaluated immediate effects; 1 did not provide post-intervention results; 7 did not measure the desired outcomes, and in 10, the experimental group did not perform an additional training). Twenty studies were finally included in the systematic review [26,29,30,38,39,40,41,42,43,44,45,46,47,48,49,50,51,52,53,54]. One of the articles [47] had the same sample as a previously published article that was also included in the present work [43]. In order to avoid duplicated samples, it was only counted once. The most recent article [47] was only included in the gait performance outcome, which was the new outcome that was added, with respect to the prior article. Furthermore, it is important to note that five of the studies included [29,42,51,52,53] were published in predatory journals. Nevertheless, characteristics of these studies matched the inclusion criteria of this systematic review and, thus, they were kept for the subsequent analyses. The participants’ mean age was 60 ± 11 years. The training program duration ranged between 2 and 8 weeks, in which 4 weeks was the most common (12 studies). The duration of each session ranged between 10 and 60 min, with 30 min being the most common duration. The total volume ranged between 240 and 1200 min, with an average of 511 min. Regarding the period of time in which the intervention started after stroke-onset, it ranged from 15 days to 34 months. The characteristics of the studies can be seen more in detail in Table 1.

### 3.1. Risk of Bias and Quality of Evidence

The studies presented poor to good methodological quality (poor: 5.3%, fair: 10.5%, good: 84.2%); (scores are presented in Table 1 and in higher detail in Table S3). The quality of the evidence was very low and low for the outcomes registered (Table S4).

### 3.2. General Effects

The effect of ATEP was assessed on trunk function, balance ability, gait performance, and functional ability. Pooled effect sizes of each outcome are shown in Table 2 and Table 3. Forest plots are also available in Figures S1–S5.

- Trunk function was evaluated in thirteen studies [38,39,40,41,42,43,45,48,49,50,52,53,54], ATEP improved trunk function by SMD 1.06 (95% CI, 0.74–1.37; I^2^ = 53%), representing a 13% of pre-post change respect to the control group.
- Balance ability was evaluated in nine studies [39,41,44,45,46,48,50,52,54], ATEP improved balance ability by SMD 0.83 (95% CI, 0.52–1.14; I^2^ 42%), which was a 17% of pre-post change respect to the control group. Balance was also assessed through the limits of stability. The forward non-affected-arm reach was analyzed in six studies [40,41,43,45,51,53], and showed that ATEP improved by SMD 0.90 (95% CI 0.47–1.33; I^2^ 43%). The lateral non-affected-arm reach was analyzed in four studies [26,40,43,53] and improved by SMD 1.16 (95% CI, 0.67–1.66; I^2^ 26%). Lastly, the lateral affected-arm reach was analyzed in three studies [40,43,53] and improved by SMD 0.89 (95% CI 0.26–1.52; I^2^ 39%).
- Gait performance was evaluated in eight studies [29,30,39,41,46,47,48,54], ATEP improved gait performance by SMD 0.63 (95% CI 0.38–0.89; I^2^ 0%).
- Functional mobility was evaluated in six studies through the TUG [29,38,41,43,44,46], ATEP improved the TUG by MD 3.40 s (95% CI, −0.32–7.12; I^2^ 67%).

### 3.3. Potential Effect Modifiers

The potential effect of the initial trunk impairment, participants’ age, the start of rehabilitation after stroke-onset, and total volume of training after ATEP was explored on trunk function, balance ability, gait performance, and functional ability. Pooled effect sizes of each potential effect modifier are shown in Table 4. Forest plots are also available in Figures S6–S9.

#### 3.3.1. Initial Trunk Impairment

The median score was 55.15% of the total score on the trunk function scale, with seven studies below the median and six studies over the median. Subgroup analyses showed high pooled effect sizes on trunk function, balance ability, and limits of stability (forward reach of the unaffected arm) for those studies with higher initial trunk impairment (1.10 < SMD < 1.54). In case of the studies with lower trunk impairment, they showed medium pooled effect sizes on the same outcomes (0.51 < SMD < 0.76). It must be noted that those participants who had a higher initial trunk impairment were older and they also started the rehabilitation programs earlier (Table 2).

#### 3.3.2. Participants Age

The median score was 58.65 years, with nine studies below and 10 studies over the median. The subgroup analyses showed high pooled effect sizes on trunk function and on limits of stability (SMD 1.13 and 1.06 respectively), and a medium effect on balance ability for the studies with older participants (SMD 0.79). In the case of the studies with younger participants, high pooled effect sizes were observed on trunk function and balance ability (SMD 0.98 and 1.12, respectively), and a medium effect on limits of stability (SMD 0.80). The change on functional mobility was higher in the older participant’s group (5.72 s vs. 1.93 s), although the change in the older group was not significant.

#### 3.3.3. Time since Stroke-Onset until Rehabilitation

The median score was 194.67 days from stroke-onset until the rehabilitation started, with nine studies below and 10 studies over the median. Subgroup analyses showed medium-to-high pooled effect sizes on trunk function, balance ability, and gait performance for those studies that started the ATEP sooner after the stroke onset (0.76 < SMD < 1.13). In the case of the studies that started the ATEP later, they also showed medium-to-high effect sizes, although the score was lower (0.59 < SMD < 0.98).

#### 3.3.4. Total Volume of Additional Trunk Exercises Program

The median score was 387.5 min, with nine studies below and nine studies over the median. The subgroup analyses showed high pooled effect sizes on trunk function, limits of stability, and gait performance for those studies with a shorter duration of ATEP (0.87 < SMD < 1.09), and a medium effect on balance ability (SMD 0.70). In the case of the studies with longer duration of ATEP, they showed high pooled effect sizes on trunk function and balance ability (SMD 1.24 and 0.95, respectively), and a low-to-medium effect on gait performance and limits of stability (SMD 0.39 and 0.72, respectively). Functional mobility improved slightly more in the shorter ATEP group (3.62 vs. 2.41 s), although, in neither of the groups was the effect significant.

## 4. Discussion

The aim of the following systematic review was to analyze how different potential effect modifiers modulate the effectiveness of trunk exercises added to conventional stroke rehabilitation programs. Firstly, the results of the present review confirmed the positive effect that ATEP have on the recovery of trunk function, balance ability, gait performance, and functional mobility. Additionally, the potential effect modifiers analyzed seemed to modulate the effectiveness of ATEP in stroke motor recovery, and should be considered when designing this type of rehabilitation programs.

### 4.1. General Effects of ATEP

Our results confirmed prior evidence [8,9,10,11] regarding the positive effect of ATEP on trunk function recovery by SMD 1.06 (CI 0.74–1.37) and balance ability (SMD 0.83; CI 0.52–1.14). The improvement on trunk function caused by ATEP represented a 13% higher than the improvement showed by the conventional therapy alone, which is equal to 3 points on the Trunk Impairment Scale (TIS). On the other hand, balance ability increased by 17%, which corresponds to a change of 9.52 points in the Berg Balance Scale. This information can be useful for practitioners as an improvement score reference when applying ATEP for trunk function and balance restoration in stroke patients. Regarding balance assessed through tests compromising the limits of stability, results also showed high effect in both the lateral reach of the affected (SMD 0.89; CI 0.26–1.52) and the non-affected arm (SMD 1.16; CI 0.67–1.66), and in the forward reach of the unaffected arm (SMD 0.90; CI 0.47–1.33). Thus, although it has been formerly said that lateral balance is more affected after a stroke [55], ATEP seem to provide the same improvement independently of the direction and of the arm involved.

In the same way, our results confirmed that ATEP improved gait performance by SMD 0.63 (CI 0.38–0.89), which supports the fact that a proper control of the trunk is a key factor to maintain balance during dynamic actions, such as gait [56]. However, contrary to what was expected, ATEP did not show a significant effect on the TUG (MD = 3.40 s; 95% CI −0.32–7.12), in spite of being a task in which the trunk control seems to play an important role because of its high balance demands [57]. The non-significant effect of ATEP on functional mobility could be caused by the fact that TUG performance depends on several parameters that could have also been impaired after a stroke, such as muscle strength in the lower limbs [58] or even cognitive or sensory deficits, hindering the ability to perform, for example, a 180° turn [59]. Nevertheless, from the authors’ point of view, observing the magnitude of the pooled effect size (3.40 s), we think that the lack of significant effect is caused by the limited number of studies analyzing this parameter. Therefore, more experimental studies are needed to confirm if ATEP have a real impact on functional mobility.

### 4.2. Effect of the Potential Effect Modifiers on Trunk Function, Balance Ability, Gait Performance, and Functional Mobility

Motor recovery after a stroke is a multifactorial process in which the interaction between different factors determines the success of a rehabilitation program [12]; however, the way in which different factors modulate the effects of ATEP has been little studied. Our results seem to indicate that a higher initial trunk impairment is related with a greater motor recovery, which can be observed not only in trunk function, but also in balance and limits of stability. These results are not in line with previous findings indicating that the more severe the motor impairment after stroke-onset, the more severe the chronic deficits [12]. A similar controversy regarding patients’ age was found, in which older age has been identified as a determinant factor for a poorer recovery process. Thirteen subgroup analyses hint that older participants would have greater improvements after ATEP for all the motor recovery parameters, except for balance. Interestingly, as Table 2 shows, those studies with older participants also displayed higher initial trunk impairment. Although, the rationale behind these findings is not clear, as mentioned elsewhere [60], the influence of initial trunk impairment and age on motor recovery after ATEP could be related with the larger room for improvement that these older or more affected patients may have. However, from the authors’ point of view, the interpretation of these findings can be biased by the fact that, in those studies with more impaired and older participants, these patients were also the ones who started the intervention after the stroke earlier. In this sense, subgroup analyses showed that the pooled effect size was slightly higher in trunk function, balance, and gait performance when stroke patients started the ATEP earlier. In spite of it being plausible that the most suitable time to start rehabilitation will also depend on the type of therapy performed, the findings of the present systematic review support the idea that ATEP would be advisable in the first stages after stroke-onset. Finally, regarding the total volume of training, it is interesting to note that longer training programs were more effective on trunk function and balance. However, shorter training programs showed better results on limits of stability, gait performance, and functional mobility. Although the controversy in these results can be caused by the low number of studies included, the results obtained seem to indicate that even short trunk training programs (<387.5 min) could be enough to induce meaningful improvements on motor recovery. Nonetheless, future research is required in order to understand the dose-response relationship of ATEP in stroke patients better.

### 4.3. Limitations

The conclusions of this systematic review should be taken with caution, as some limitations are present. First, the main limitation regards the non-statistical comparison between subgroups for the potential effect modifiers analyzed. Although interesting information has been advertised from the subgroups results, they have to be interpreted with caution since they have not compared statistically. Likewise, the quality of evidence obtained with the GRADE approach was low and very low for all outcomes analyzed (Table S4, Supplementary Material). Therefore, higher quality of evidence is needed to reinforce or reject the findings of the present work. In addition, not all of the studies assessed all of the outcomes and, thus, there was a lower sample in some of the variables registered, especially, when subgroup analyses were carried out to assess the impact of the potential effect modifiers. In the same way, as not all of the characteristics were provided in all of the articles, it was not possible to perform the same subgroup analyses of all potential effect modifiers for all the studies included. Regarding the training volume, it must be noted that the experimental group performed a higher total intervention volume, which is an intrinsic feature of any supplementary program added to the conventional therapy. However, it would be interesting that future studies compare the effectiveness of trunk exercise programs versus conventional rehabilitation, equaling the intervention volume in both groups to obtain a clearer view about time cost–benefit of each program. Furthermore, it would also be interesting to analyze different types of additional exercise programs to compare which of them are more effective in stroke rehabilitation. Finally, although ratio-scales allow a quick and easy-to-use evaluation of several parameters, future studies would need to implement tests that employ more quantifiable and objective parameters to assess the different capabilities. For example, following Veerbeek et al.’s proposal [61], the implementation of wearable devices, such as accelerometers, would be helpful to objectively quantify motor recovery parameters.

## 5. Conclusions

The results of the present systematic review confirmed the positive effects on trunk function, balance ability, gait performance, and functional mobility recovery when trunk exercises were added to the conventional rehabilitation therapy. Regarding the potential effect modifiers analyzed (i.e., initial trunk impairment, age, intervention onset, and ATEP training volume), it seems that these might play an important role in the modulation of ATEP. Older patients, and those with higher initial trunk impairment, obtained, in general, greater improvements on the outcomes assessed. Moreover, it is important to note that these patients were also those who started the rehabilitation program earlier, which was also linked with a larger motor recovery. Regarding the volume of the ATEP, it seems that short durations could be enough to cause positive effects on motor recovery. Thus, since potential effect modifiers seem to modulate ATEP effectiveness, future studies should consider them to better understand their impact in stroke rehabilitation. Finally, the quality of the evidence was low, and thus, higher quality studies are required in order to strengthen evidence towards ATEP in rehabilitation programs after stroke.

## Figures and Tables

**Table 1:** Characteristics of the studies included in the systematic review.

General Characteristics						Training Programs Characteristics					PEDro Scale
Study	N Total (EG, CG)	Age (Mean ± SD)	Time after Stroke Months [days]	Outcomes	Intervention	Program	Total Weeks	SESSIONS PER WEEK	Duration (min)	Total Duration (min)	
DeSèze et al., 2001	20 (10, 10)	EG(63.5 ± 17.0) CG(67.7 ± 15.0)	1.1 [32.3]	Trunk, control test, Equilibrium Index, FAC	**EG**: CRP + trunk control training pointing targets (through a device located above the head) with auditory and visual feedback when the target is reached. They performed weight shifting of the trunk in many directions.	ATEP	4	5	60	1200	8 Good Quality
						ATEP +CRP	12	10–5	180–120	6000	
					**CG**: CRP (Bobath-inspired approach + functional therapy).	CRP	8	5	120	4800	
Howe et al., 2005	33 (15, 18)	EG(71.5 ± 10.9) CG(70.7 ± 7.9)	0.8 [24.8]	LRT (standing)	**EG**: CRP + trunk weight transference in sitting and standing position. Movements involved trunk flexion and extension, lateral flexion, moving objects from the unaffected to the affected arm beyond the base of support. They also performed exercises changing from sitting to standing position.	ATEP	4	3	30	360	6 Good Quality
						ATEP +CRP	4	NS	NS	7495	
					**CG**: CRP (Usual care and physiotherapy).	CRP	4	NS	NS	8643	
Dean et al., 2007	12 (6, 6)	EG(60.0 ± 7.0) CG(74.0 ± 12.0)	0.9 [29.0]	10 m walk test	**EG**: CRP + lateral trunk weight transference in sitting position. Movements included trunk frontal and lateral flexion and extension, reaching for objects of different weight placed at different points on a table.	ATEP	2	5	30	300	8 Good Quality
						ATEP +CRP	2	10	60	600	
					**CG**: CRP + sham sitting training	CRP	2	5	30	300	
Verheyden et al., 2009	33 (17, 16)	EG(55.0 ± 11.0) CG(62.0 ± 14.0)	1.7 [51.0]	TIS	**EG**: CRP + trunk exercises in supine position: legs bent and feet on the table, they performed anteroposterior pelvic movements, back bridge, and upper and lower trunk rotation; and in sitting position: weight transference involving trunk and hip flexion and extension movements.	ATEP	5	4	30	600	8 Good Quality
						ATEP +CRP	5	NS	NS	NS	
					**CG**: CRP (physiotherapy, occupational therapy, nursing care).	CRP	5	NS	NS	600	
Yoo et al., 2010	59 (28, 31)	EG(59.6 ± 18.1) CG(61.7 ± 12.5)	1.5 [45.5]	TIS, BBS	**EG**: CRP + trunk exercises divided in 3 levels based on the difficulty level (1: trunk bracing, bridge exercise, segmental rotation; 2: dead bug, hamstring curls, crossed extension; 3: side bridge, belly blaster, and the bird-dog exercise).	ATEP	4	3	30	360	3 Poor Quality
						ATEP+CRP	4	3	NS	NS	
					**CG**: CRP (neurodevelopment treatment, walking, occupational therapy).	CRP	4	3	NS	NS	
Kim et al., 2011	40 (20, 20)	EG(51.4 ± 5.7) CG(53.5 ± 7.1)	24.8 [755.9]	FRT (standing)	**EG**: CRP + trunk exercises with proprioceptive neuromuscular facilitation (stabilizing reversal and rhythmic stabilization techniques) in sitting and standing positions.	ATEP	6	5	10	300	5 Fair Quality
						ATEP +CRP	6	10	40	1200	
					**CG**: CRP (Stretching and Range of Movement exercises).	CRP	6	5	30	900	
Vijayakumar et al., 2011	20 (10,10)	EG(59.5 ± 12.9) CG(57.8 ± 13.4)	0.5 [15.4]	TIS, BBA	**EG**: CRP + trunk exercises in supine position: back bridge, unilateral pelvic bridge, upper and lower trunk rotation; and sitting position: static sitting balance, trunk flexion and extensions, trunk lateral flexion.	ATEP	3	6	45	810	5 Fair Quality
						ATEP +CRP	3	NS	NS	NS	
					**CG**: CRP (Usual care including physiotherapy).	CRP	3	NS	NS	NS	
Lee et al., 2011	28 (14, 14)	EG(59.0 ± 11.0) CG(62.3 ± 14.2)	34.0 [1034.2]	TIS, mFRT (sitting forward and both sides lateral reach)	**EG**: CRP + dual motor task in sitting position (trunk weight transference). Patients sat on an unstable seat with hips and knees 90° flexed performing movements on the frontal plane. They also threw a ball to targets and afterwards did fishing and played badminton while sitting on the unstable surface.	ATEP	6	3	30	540	6 Good Quality
						ATEP +CRP	6	8	90	2340	
					**CG**: CRP (Brunnstrom motion therapy, Bobath neurological development, and PNF).	CRP	6	5	60	1800	
Saeys et al., 2012	33 (18, 15)	EG(61.9 ± 13.8) CG(61.0 ± 9.0)	1.2 [35.4]	TIS, BBS, FAC	**EG**: CRP + trunk muscle exercises in supine position: back bridges, shoulder girdle lifts (symmetrical and asymmetrical); and sitting position: anteroposterior pelvic tilt, upper and lower trunk rotation, reaching tasks beyond arm’s length, shuffling forward and backwards, sitting on unstable seat.	ATEP	8	4	30	960	8 Good Quality
						ATEP +CRP	8	8	60	1920	
					**CG**: CRP (physical and occupational therapy) + passive mobilization of upper limb and transcutaneous electrical nerve stimulation of the hemiplegic shoulder.	CRP	8	4	30	960	
Chung et al., 2013	16 (8, 8)	EG(44.3 ± 9.9) CG(48.3 ± 9.7)	11.3 [342.3]	TUG, 3 m walk test	**EG**: CRP + core stability exercises consisting in three parts: 1) bed exercises (bridge, bridge with legs crossed, unipedal back bridge), 2) wedge exercises (forward and lateral curl-ups, bird-dog exercise and side bridge); 3) ball exercises (bridge, bridge to side, bridge-up, curl-ups, bird-dog exercise and push-ups).	ATEP	4	3	30	360	6 Good Quality
						ATEP +CRP	4	8	90	1560	
					**CG**: CRP (stretching, strengthening and stationary bicycle).	CRP	4	5	60	1200	
Jung et al., 2014	17 (9, 8)	EG(51.9 ± 10.3) CG(57.9 ± 8.5)	14.4 [437.2]	TIS, TUG	**EG**: CRP + weight-shifting training in two sitting positions (knees extended on a mat, and knees flexed on the edge of a table). Exercises involved static sitting balance (unstable seat) and trunk movements forward, backwards and in lateral directions.	ATEP	4	5	30	600	6 Good Quality
						ATEP +CRP	4	10	60	1200	
					**CG**: CRP (physiotherapy, stretching, strengthening, stationary bike).	CRP	4	5	60	1200	
Cabanas-Valdés et al., 2015	79 (40, 39)	EG(74.9 ± 10.7) CG(75.6 ± 9.4)	0.8 [23.6]	TIS 2.0, BBS, Tinetti scale (gait subscale)	**EG**: CRP + core stability exercises in supine position: pelvis anteversion and retroversion, back bridge, unilateral back bridge with the unaffected leg, upper and lower trunk rotation, back bridge (bilateral and unilateral) with Swiss ball; and sitting position (on stable and unstable surfaces): trunk flexion and extension, lateral trunk flexion starting from the shoulders, upper and lower trunk rotation, and forward reach in three directions.	ATEP	5	5	15	375	7 Good Quality
						ATEP +CRP	5	10	75	1875	
					**CG**: CRP (physiotherapy, walking, occupational therapy, nursing care)	CRP	5	5	60	1500	
Jung et al., 2015	22 (11, 11)	EG(53.1 ± 16.6) CG(54.1 ± 9.1)	16.1 [490.8]	TIS, FRT (sitting forward and both sides lateral reach)	**EG**: CRP + core stability training with visual feedback of the center of pressure while sitting on an unstable seat (trunk and pelvis movements).	ATEP	4	3	20	240	6 Good Quality
						ATEP +CRP	4	8	80	1440	
					**CG**: CRP (Brunnstrom approach exercise, neuro-development treatment with Bobath concepts, neuromuscular facilitation).	CRP	4	5	60	1200	
Haruyama et al., 2016	32 (16, 16)	EG(67.5 ± 10.1) CG(65.6 ± 11.9)	2.3 [69.0]	TIS, Brief BESTest, FRT (standing), FAC, TUG	**EG**: CRP + core stability training with abdominal hollowing: pelvic control exercises in supine and sitting positions. They performed anteroposterior tilt, lateral lift, and transverse rotation.	ATEP	4	5	20	400	7 Good Quality
						ATEP +CRP	4	10	80	1600	
					**CG**: CRP (physical therapy, occupational therapy, speech therapy, nursing care, bridges, pelvic movements and reaching exercises).	CRP	4	5	60	1200	
Shin et al., 2016	24 (12, 12)	EG(57.7 ± 14.0) CG(59.2 ± 9.7)	16.4 [498.4]	TIS, FRT (sitting forward and both sides lateral reach), TUG. 10 m walk test* (Data from Shin, 2020)	**EG**: CRP + core stability training in sitting position with an unstable seat that tilts in any direction and provides visual feedback. Patients moved their center of pressure to point at the target through pelvic and trunk movements.	ATEP	4	3	20	240	8 Good Quality
						ATEP +CRP	4	8	100	1840	
					**CG**: CRP (physical and occupational therapy and electrical stimulation therapy).	CRP	4	5	80	1600	
Rose et al., 2016	24 (12, 12)	EG(57.0 ± 2.8) GC(56.7 ± 3.1)	6.4 [194.7]	TIS	**EG**: CRP + core stability training in prone position: trunk extension; and sitting position: trunk flexion from sitting reclined position (120°), leaning back and forward, from sitting position with trunk rotated to the hemiplegic side the patient lies down, and lateral flexion of the trunk.	ATEP	4	3	NS	NS	7 Good Quality
						ATEP +CRP	4	NS	NS	NS	
					**CG**: CRP (Usual care including physiotherapy).	CRP	4	NS	NS	NS	
An et al., 2017	29 (15, 14)	EG(59.7 ± 8.9) CG(57.1 ± 17.1)	9.1 [277.9]	BBS, TUG	**EG**: CRP + trunk exercises in supine position: back bridge, unilateral back bridge, upper and lower trunk rotation; and sitting position: flexion and extension of the lower trunk, upper and lower trunk lateral flexion, upper and lower trunk rotation, forward and lateral reach.	ATEP	4	3	30	360	6 Good Quality
						ATEP +CRP	4	8	60	960	
					**CG**: CRP (neurodevelopment therapy).	CRP	4	5	30	600	
Park et al. 2019	28 (14,14)	EG(56.2 ± 13.7) CG(57.1 ± 11.7)	12 [336.7]	TIS, BBS-3L, FRT (standing)	º	ATEP	4	5	30	600	6 Good Quality
						ATEP +CRP	4	10	90	1800	
					**CG**: CRP (neurodevelopment treatment Bobath approach).	CRP	4	5	60	1200	
Min et al., 2020	38 (19, 19)	EG(61.4 ± 11.1) CG(56.3 ± 9.1)	28.1 [855.1]	BBS, 10 m walk test, TUG	**EG**: CRP + trunk stability training with a robot system (standing balance, sitting balance and move from sitting to standing).	ATEP	4	5	30	600	8 Good Quality
						ATEP +CRP	4	10	60	1200	
					**CG**: CRP (symmetrical static and dynamic standing balance during walking).	CRP	4	5	30	600	

SD: Standard Deviation; EG: Experimental Group; CG: Control Group; TIS: Trunk Impairment Scale; BBS: Berg Balance Scale; BBS–3L: 3-level Berg Balance Scale; BBA: Brunel Balance Assessment; TUG: Timed Up and Go; FRT: Functional Reach Test; LRT: Lateral Reach Test; FAC: Functional Ambulation Categories; CRP: Conventional Rehabilitation Program; NS: non-specified.

**Table 2:** Pooled effect size in trunk function of additional trunk exercises vs. conventional rehabilitation and potential effect modifiers characteristics depending on the initial trunk impairment.

	N (Studies)	N (Sample)	SMD	LCL	UCL		I^2^	*p*
Trunk function	13	419	1.06	0.74	1.37		53	<0.01
	Studies with higher initial trunk impairment		Studies with lower initial trunk impairment					
	Mean	(SD)	Mean			(SD)		
Initial trunk impairment (%)	43.6	(11.1)	66.5			(7.0)		
Stroke-onset (days)	240.7	(391.1)	263.2			(187.0)		
Total volume of additional trunk exercises (min)	582.9	(319.7)	440.0 *			(157.5)*		
Participants’ age	62.3	(5.9)	56.8			(5.6)		

* Rose et al. (2016) was not included because it did not provide the total volume of training. SMD: standardized mean difference; LCL: lower confidence limit; UCL: upper confidence limit; I^2^ statistic (%): heterogeneity statistic. The effect was in favor of additional trunk exercises when the SMD is positive.

**Table 3:** Pooled effect sizes in balance, limits of stability, gait performance, and functional mobility of additional trunk exercises vs. conventional rehabilitation.

	N (Studies)	N (Sample)	SMD	LCL	UCL	I^2^	*p*
Balance ability	9	338	0.83	0.52	1.14	42	<0.01
LOS forward unaffected arm	6	174	0.90	0.47	1.33	43	<0.01
LOS lateral unaffected arm	4	107	1.16	0.67	1.66	26	<0.01
LOS lateral affected arm	3	74	0.89	0.26	1.52	39	<0.01
Gait performance	8	254	0.63	0.38	0.89	0	<0.01
Functional mobility	6	156	3.40 *	−0.32	7.12	67	0.07

* Pooled effect size was obtained through the weighted mean difference since all of the studies employed the same test/scale. SMD: standardized mean difference; LCL: lower confidence limit; UCL: upper confidence limit; I^2^ (%): heterogeneity statistic; LOS: limits of stability. The effect was in favor of additional trunk exercises when the SMD is positive.

**Table 4:** Pooled effect sizes on the outcomes sub-grouped by the potential effect modifiers.

Initial Impairment (Median of the Studies 55.15% of the Trunk Function Pre-Test Score)														
**Outcomes**	Studies with higher trunk impairment							Studies with lower trunk impairment						
	**Initial trunk impairment (%)**		**Total** **N**	**SMD**	**LCL**	**UCL**	**I^2^**	**Initial trunk impairment (%)**		**Total N**	**SMD**	**LCL**	**UCL**	**I^2^**
	Mean	(SD)						Mean	(SD)					
Trunk function	43.6	11.1	252	1.32	0.87	1.78	56	66.5	7.0	156	0.76	0.40	1.12	15
Balance ability	43.0	12.2	211	1.10	0.51	1.70	71	67.4	0.1	60	0.65	0.13	1.17	0
LOS–forward reach	45.3	11.5	52	1.54	0.91	2.18	0	70.8	5.8	82	0.51	0.06	0.95	0
**Participants’ age (median of the studies 58.65 Years)**														
**Outcomes**	Younger participants							Older participants						
	**Participants’ age (years)**		**Total** **N**	**SMD**	**LCL**	**UCL**	**I^2^**	**Participants’ age (years)**		**Total** **N**	**SMD**	**LCL**	**UCL**	**I^2^**
	Mean	(SD)						Mean	(SD)					
Trunk function	56.5	2.0	144	0.98	0.35	1.61	66	64.1	5.7	275	1.13	0.79	1.46	37
Balance ability	57.9	1.1	77	1.12	0.06	2.18	77	64.8	5.9	261	0.79	0.53	1.05	3
LOS-forward reach	54.2	2.2	90	0.80	0.37	1.24	0	61.9	4.2	84	1.06	0.11	2.01	75
Functional mobility	53.2	6.2	62	1.93 *	0.10	3.76	0	61.3	4.6	94	5.72 *	−2.27	13.72	40
**Time from stroke-onset until the start of the rehabilitation program (median of the studies 194.67 days)**														
	Studies starting the rehabilitation program earlier							Studies starting the rehabilitation program later						
**Outcomes**	**Time after stroke-onset (days)**		**Total N**	**SMD**	**LCL**	**UCL**	**I^2^**	**Time after stroke-onset (days)**		**Total N**	**SMD**	**LCL**	**UCL**	**I^2^**
	Mean	(SD)						Mean	(SD)					
Trunk function	38.8	18.0	276	1.13	0.65	1.61	67	498.7	286.1	143	0.98	0.55	1.41	31
Balance ability	36.8	18.8	243	0.98	0.52	1.44	60	499.9	310.8	95	0.62	0.21	1.03	0
Gait performance	38.4	20.8	143	0.76	0.41	1.10	0	565.3	262.9	78	0.59	−0.08	1.26	48
**Total volume of additional trunk exercises program (median of the studies 387.5 min)**														
**Outcomes**	Studies with lower volume of additional trunk exercises							Studies with higher volume of additional trunk exercises						
	**ATEP (min)**		**Total** **N**	**SMD**	**LCL**	**UCL**	**I^2^**	**ATEP (min)**		**Total** **N**	**SMD**	**LCL**	**UCL**	**I^2^**
	Mean	(SD)						Mean	(SD)					
Trunk function	303.8	73.9	184	0.97	0.58	1.35	29	696.9	257.4	211	1.24	0.80	1.69	52
Balance ability	365.0	8.7	167	0.70	0.21	1.19	55	739.2	289.5	171	0.95	0.50	1.39	44
LOS-forward reach	260.0	34.6	86	1.09	0.63	1.55	0	513.3	102.6	88	0.72	−0.03	1.47	65
Gait performance	318.8	61.7	131	0.87	0.51	1.23	0	790.0	358.3	123	0.39	0.03	0.75	0
Functional mobility	320.0	69.3	69	3.62 *	−0.78	8.01	84	533.3	115.5	87	2.41 *	−6.29	11.11	23

* Pooled effect size was obtained through the weighted mean difference since all the studies employed the same test/scale. SMD: Standardized mean difference; LCL: lower confidence limit; UCL: upper confidence limit; I^2^ (%): heterogeneity statistic; ATEP: Additional trunk exercises program. The effect was in favor of additional trunk exercises when the SMD is positive.

## Supplementary Materials

The following are available online at https://www.mdpi.com/1660-4601/17/23/8714/s1, Figure S1: Pooled effect sizes on trunk function; Figure S2: Pooled effect sizes on balance ability; Figure S3: Pooled effect sizes on limits of stability; Figure S4: Pooled effect sizes on gait performance; Figure S5: Pooled effect sizes on functional mobility; Figure S6:Subgroup analyses by initial trunk impairment; Figure S7: Subgroup analyses by participants’ age; Figure S8: Subgroup analyses by the start of the intervention after stroke-onset; Figure S9: Subgroup analyses by total volume (minutes) of the additional trunk exercise programs, Table S1: PRISMA checklist; Table S2: Boolean search strategy for each database; Table S3: PEDro scale to assess methodological quality; Table S4: Quality of evidence (GRADE approach) between additional trunk-focused exercises vs. conventional rehabilitation.
