# Supplementary material for: Do Initial Trunk Impairment, Age, Intervention Onset, and Training Volume Modulate the Effectiveness of Additional Trunk Exercise Programs after Stroke? A Systematic Review with Meta-Analyses
Source: Int J Environ Res Public Health. 2020 Nov 24;17(23):8714. doi: 10.3390/ijerph17238714 (PMC7727690; doi:10.3390/ijerph17238714)
Supplement: Supplementary file 1 [file ijerph-17-08714-s001.pdf]

Supplementary material

Table S1. PRISMA checklist.

Section/topic	#	Checklist item
**TITLE**		
Title	1	Identify the report as a systematic review, meta-analysis, or both.
**ABSTRACT**		
Structured summary	2	Provide a structured summary including, as applicable: background; objectives; da eligibility criteria, participants, and interventions; study appraisal and synthesis m limitations; conclusions and implications of key findings; systematic review registr
**INTRODUCTION**		
Rationale	3	Describe the rationale for the review in the context of what is already known.
Objectives	4	Provide an explicit statement of questions being addressed with reference to partic comparisons, outcomes, and study design (PICOS).
**METHODS**		
Protocol and registration	5	Indicate if a review protocol exists, if and where it can be accessed (e.g., Web addre provide registration information including registration number.
Eligibility criteria	6	Specify study characteristics (e.g., PICOS, length of follow-up) and report characte considered, language, publication status) used as criteria for eligibility, giving ratio
Information sources	7	Describe all information sources (e.g., databases with dates of coverage, contact wi identify additional studies) in the search and date last searched.
Search	8	Present full electronic search strategy for at least one database, including any limit could be repeated.
Study selection	9	State the process for selecting studies (i.e., screening, eligibility, included in system applicable, included in the meta-analysis).
Data collection process	10	Describe method of data extraction from reports (e.g., piloted forms, independentl any processes for obtaining and confirming data from investigators.
Data items	11	List and define all variables for which data were sought (e.g., PICOS, funding sour assumptions and simplifications made.
Risk of bias in individual studies	12	Describe methods used for assessing risk of bias of individual studies (including sp whether this was done at the study or outcome level), and how this information is synthesis.
Summary measures	13	State the principal summary measures (e.g., risk ratio, difference in means).
Synthesis of results	14	Describe the methods of handling data and combining results of studies, if done, ir consistency (e.g., I^2) for each meta-analysis.
Risk of bias across studies	15	Specify any assessment of risk of bias that may affect the cumulative evidence (e.g. selective reporting within studies).

Additional analyses	16	Describe methods of additional analyses (e.g., sensitivity or subgroup analyses, me indicating which were pre-specified.
**RESULTS**		
Study selection	17	Give numbers of studies screened, assessed for eligibility, and included in the revie exclusions at each stage, ideally with a flow diagram.
Study characteristics	18	For each study, present characteristics for which data were extracted (e.g., study si period) and provide the citations.
Risk of bias within studies	19	Present data on risk of bias of each study and, if available, any outcome level asses
Results of individual studies	20	For all outcomes considered (benefits or harms), present, for each study: (a) simple each intervention group (b) effect estimates and confidence intervals, ideally with a
Synthesis of results	21	Present results of each meta-analysis done, including confidence intervals and mea
Risk of bias across studies	22	Present results of any assessment of risk of bias across studies (see Item 15).
Additional analysis	23	Give results of additional analyses, if done (e.g., sensitivity or subgroup analyses, r Item 16]).
**DISCUSSION**		
Summary of evidence	24	Summarize the main findings including the strength of evidence for each main out relevance to key groups (e.g., healthcare providers, users, and policy makers).
Limitations	25	Discuss limitations at study and outcome level (e.g., risk of bias), and at review-lev retrieval of identified research, reporting bias).
Conclusions	26	Provide a general interpretation of the results in the context of other evidence, and research.
**FUNDING**		
Funding	27	Describe sources of funding for the systematic review and other support (e.g., supp funders for the systematic review.

From: Moher D, Liberati A, Tetzlaff J, Altman DG, The PRISMA Group (2009). Preferred Reporting Items for Systematic Reviews and Meta-Analyses: The PRISMA Statement. PLoS Med 6(6): e1000097. doi:10.1371/journal.pmed1000097

For more information, visit: **www.prisma-statement.org**.

Table S2. Boolean search strategy for each database.

**PubMed**	(("core strength"[Title/Abstract] OR "trunk strength"[Title/Abstract] OR "trunk stability"[Title/Abstract] OR "trunk stabilization"[Title/Abstract] OR "trunk control"[Title/Abstract] OR "core stability"[Title/Abstract] OR "core stabilization"[Title/Abstract] OR "core control"[Title/Abstract] OR "lumbar stability"[Title/Abstract] OR "lumbar stabilization"[Title/Abstract] OR "lumbar control"[Title/Abstract] OR "spine stability"[Title/Abstract] OR "spine stabilization"[Title/Abstract] OR "spine control"[Title/Abstract] OR "lumbopelvic stability"[Title/Abstract] OR "lumbopelvic control"[Title/Abstract] OR "lumbopelvic stabilization"[Title/Abstract] OR "lumbo-pelvic stability"[Title/Abstract] OR "lumbo-pelvic control"[Title/Abstract] OR "lumbo-pelvic stabilization"[Title/Abstract]) AND ("training"[Title/Abstract] OR "exercises"[Title/Abstract] OR "program"[Title/Abstract] OR "programme") AND ("stroke"[Title/Abstract]) NOT "cell"[Title/Abstract])
**Scopus**	TITLE-ABS ("core strength" OR "trunk strength" OR "trunk stability" OR " trunk stabilization" OR "trunk control" OR "core stability" OR "core stabilization" OR "core control" OR "lumbar stability" OR "lumbar stabilization" OR "lumbar control" OR "spine stability" OR "spine stabilization" OR "spine control" OR "lumbopelvic stability" OR "lumbopelvic control" OR "lumbopelvic stabilization" OR "lumbo-pelvic stability" OR "lumbo-pelvic control" OR "lumbo-pelvic stabilization") AND ("training" OR "exercises" OR "program" OR "programme") AND ("stroke") AND NOT ("cell") AND (LIMIT-TO (DOCTYPE , "ar")) AND (LIMIT-TO (LANGUAGE , "English"))
**Cochrane and EMBASE**	("core strength" OR "trunk strength" OR "trunk stability" OR "trunk stabilization" OR "trunk control" OR "core stability" OR "core stabilization" OR "core control" OR "lumbar stability" OR "lumbar stabilization" OR "lumbar control" OR "spine stability" OR "spine stabilization" OR "spine control" OR "lumbopelvic stability" OR "lumbopelvic control" OR "lumbopelvic stabilization" OR "lumbo-pelvic stability" OR "lumbo-pelvic control" OR "lumbo-pelvic stabilization") AND ("training" OR "exercises" OR "program" OR "programme") AND ("stroke") AND NOT ("cell")

Forest plot of the main outcomes analyzed

Study or Subgroup	Experimental Mean	SD	Total	Control Mean	SD	Total	Weight	Std. Mean Difference IV, Random, 95% CI	Year
De Sèze 2001	33.7	11.5	10	20	18.3	10	6.7%	0.86 [−0.07, 1.78]	2001
Verheyden 2009	4.82	2.69	17	3.31	3.59	16	8.9%	0.47 [−0.23, 1.16]	2009
Yoo 2010	4.78	4.55	28	2.45	4.31	31	10.9%	0.52 [−0.00, 1.04]	2010
Vijayakumar 2011	6.96	1.28	10	3.13	1.24	10	4.1%	2.91 [1.58, 4.25]	2011
Lee 2011	3.7	2.3	14	0.9	1.4	14	7.4%	1.43 [0.58, 2.27]	2011
Saeys 2012	8.72	2.8	18	2.87	3.23	15	7.4%	1.90 [1.06, 2.74]	2012
Jung 2014	2.4	1.5	9	0.1	2.38	8	5.8%	1.11 [0.07, 2.16]	2014
Jung 2015	2.36	2.94	11	−0.27	1.27	11	6.8%	1.12 [0.21, 2.03]	2015
Cabanas-Valdés 2015	5.88	3.48	40	2.5	2.2	39	11.4%	1.15 [0.67, 1.62]	2015
Rose 2016	1.83	1.28	12	1.75	2.19	12	7.8%	0.04 [−0.76, 0.84]	2016
Haruyama 2016	4.13	2.38	16	1.19	2.79	16	8.3%	1.11 [0.35, 1.86]	2016
Shin 2016	3.08	2.71	12	0.09	1.24	12	6.8%	1.37 [0.46, 2.28]	2016
Park 2019	3.07	2.04	14	0.93	2.23	14	7.9%	0.97 [0.18, 1.76]	2018
**Total (95% CI)**			**211**			**208**	**100.0%**	**1.06 [0.74, 1.37]**	

Heterogeneity: Tau² = 0.17; Chi² = 25.59, df = 12 (P = 0.01); I² = 53%
Test for overall effect: Z = 6.51 (P < 0.00001)

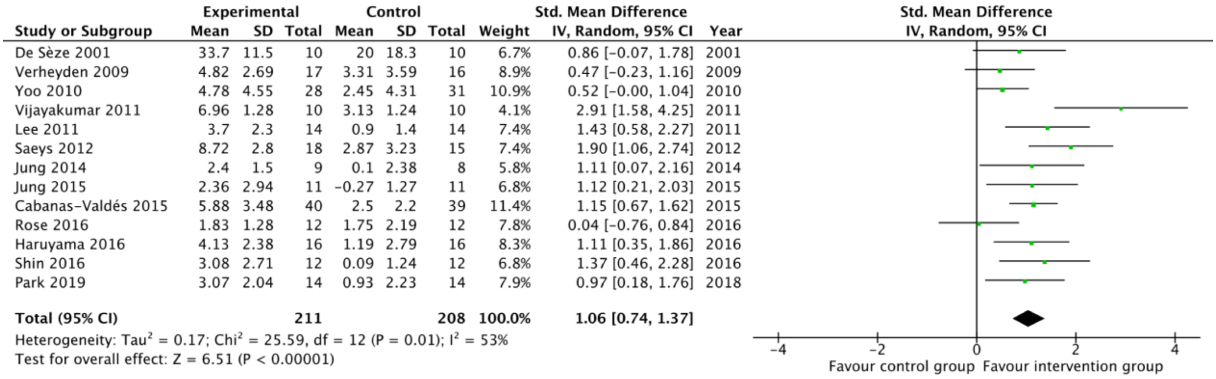

Figure S1. Pooled effect sizes on trunk function.

Study or Subgroup	Experimental Mean	SD	Total	Control Mean	SD	Total	Weight	Std. Mean Difference IV, Random, 95% CI	Year
De Sèze 2001	1.6	0.5	10	0.8	0.7	10	7.3%	1.26 [0.28, 2.24]	2001
Yoo 2010	11.29	13.25	28	6.32	11.99	31	15.6%	0.39 [−0.13, 0.91]	2010
Vijayakumar 2011	7.1	0.88	10	4.4	1.1	10	5.0%	2.60 [1.34, 3.85]	2011
Saeys 2012	19.39	12.61	18	9.2	13.52	15	11.2%	0.76 [0.05, 1.48]	2012
Cabanas-Valdés 2015	23.03	15.95	40	8.49	8.74	39	16.7%	1.12 [0.64, 1.59]	2015
Haruyama 2016	2.79	3.34	16	0.06	4.32	16	11.1%	0.69 [−0.03, 1.41]	2016
An 2017	2.93	2.96	15	1.36	2.84	14	10.6%	0.53 [−0.22, 1.27]	2017
Park 2019	4.79	3.06	14	2.86	3.19	14	10.3%	0.60 [−0.16, 1.36]	2018
Min 2020	7.36	3.63	19	4.42	4.49	19	12.3%	0.71 [0.05, 1.36]	2020
**Total (95% CI)**			**170**			**168**	**100.0%**	**0.83 [0.52, 1.14]**	

Heterogeneity: Tau² = 0.09; Chi² = 13.81, df = 8 (P = 0.09); I² = 42%
Test for overall effect: Z = 5.29 (P < 0.00001)

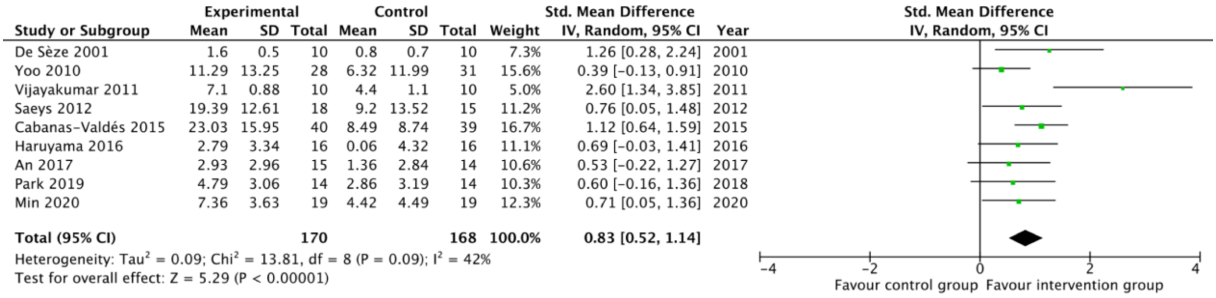

Figure S2. Pooled effect sizes on balance ability.

A)

Study or Subgroup	Experimental Mean	SD	Total	Control Mean	SD	Total	Weight	Std. Mean Difference IV, Random, 95% CI	Year
Kim 2011	4.6	5.54	20	0.1	4.1	20	20.3%	0.91 [0.25, 1.56]	2011
Lee 2011	16.5	9.7	14	4.1	5.6	14	15.2%	1.52 [0.66, 2.38]	2011
Jung 2015	8.28	5.42	11	2.37	6.15	11	14.4%	0.98 [0.09, 1.88]	2015
Haruyama 2016	3.37	4.47	16	2.28	6.49	16	19.1%	0.19 [−0.50, 0.89]	2016
Shin 2016	7.36	5.5	12	0.99	0.63	12	13.5%	1.57 [0.63, 2.51]	2016
Park 2019	5.69	6.24	14	2.71	4.21	14	17.5%	0.54 [−0.21, 1.30]	2018
**Total (95% CI)**			**87**			**87**	**100.0%**	**0.90 [0.47, 1.33]**	

Heterogeneity: Tau² = 0.12; Chi² = 8.82, df = 5 (P = 0.12); I² = 43%
Test for overall effect: Z = 4.13 (P < 0.0001)

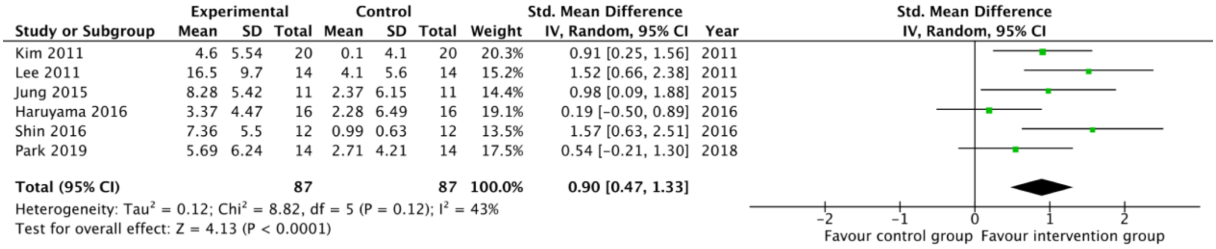

B)

Study or Subgroup	Experimental Mean	SD	Total	Control Mean	SD	Total	Weight	Std. Mean Difference IV, Random, 95% CI	Year
Howe 2005	1	1.1	15	0.4	0.59	18	32.1%	0.68 [−0.03, 1.39]	2005
Lee 2011	8.7	5.2	14	1.8	2.8	14	24.0%	1.60 [0.74, 2.47]	2011
Jung 2015	7.91	5.01	11	0.95	2.48	11	19.3%	1.69 [0.69, 2.70]	2015
Shin 2016	8.36	4.5	12	3.44	5.46	12	24.7%	0.95 [0.10, 1.80]	2016
**Total (95% CI)**			**52**			**55**	**100.0%**	**1.16 [0.67, 1.66]**	

Heterogeneity: Tau² = 0.07; Chi² = 4.08, df = 3 (P = 0.25); I² = 26%
Test for overall effect: Z = 4.63 (P < 0.00001)

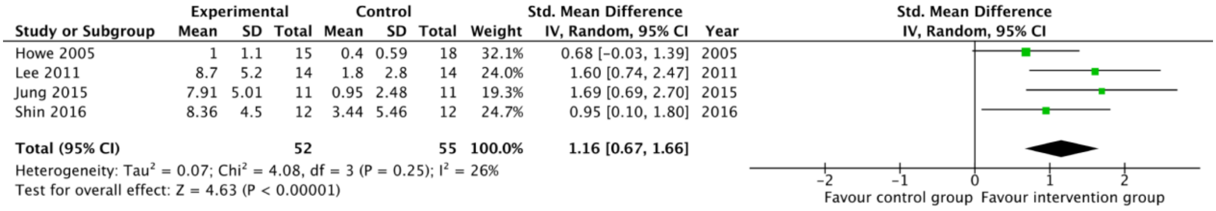

C)

Study or Subgroup	Experimental Mean	SD	Total	Control Mean	SD	Total	Weight	Std. Mean Difference IV, Random, 95% CI	Year
Lee 2011	5.3	4.4	14	3.5	2.1	14	38.2%	0.51 [−0.25, 1.26]	2011
Jung 2015	5.66	6.35	11	1.65	4.2	11	32.5%	0.72 [−0.15, 1.58]	2015
Shin 2016	6.32	3.79	12	1.7	1.13	12	29.3%	1.60 [0.65, 2.54]	2016
**Total (95% CI)**			**37**			**37**	**100.0%**	**0.89 [0.26, 1.52]**	

Heterogeneity: Tau² = 0.12; Chi² = 3.29, df = 2 (P = 0.19); I² = 39%
Test for overall effect: Z = 2.78 (P = 0.005)

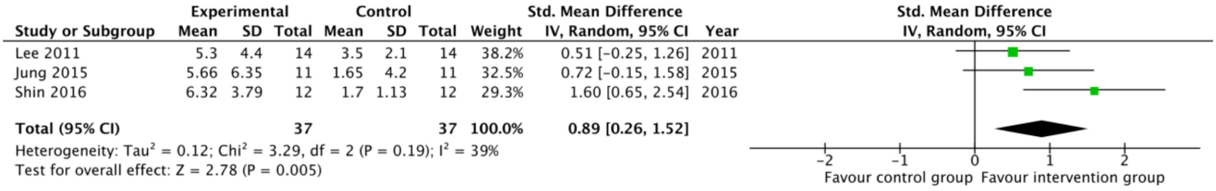

Figure S3. (A) Pooled effect sizes on limits of stability forward reach of the unaffected arm; (B) Pooled effect sizes on limits of stability lateral reach of the unaffected arm; (C) Pooled effect sizes on limits of stability lateral reach of the affected arm.

Study or Subgroup	Experimental Mean	Experimental SD	Experimental Total	Control Mean	Control SD	Control Total	Weight	Std. Mean Difference IV, Random, 95% CI	Year
De Sèze 2001	2.2	1	10	1.1	1.1	10	7.3%	1.00 [0.06, 1.94]	2001
Dean 2007	0.41	0.35	6	0.21	0.16	6	4.7%	0.68 [−0.50, 1.86]	2007
Saeys 2012	2.45	1.31	18	1.86	1.38	15	13.5%	0.43 [−0.27, 1.12]	2012
Chung 2013	14.08	13.9	8	−0.3	11.24	8	5.7%	1.08 [0.01, 2.15]	2013
Cabanas-Valdés 2015	5.57	3.97	40	2.62	2.78	39	30.5%	0.85 [0.39, 1.31]	2015
Haruyama 2016	0.5	0.81	16	0.12	0.93	16	13.2%	0.42 [−0.28, 1.13]	2016
Shin 2020	0.17	0.15	12	0.05	0.1	12	9.0%	0.91 [0.06, 1.76]	2019
Min 2020	0.36	0.58	19	0.32	0.71	19	16.1%	0.06 [−0.58, 0.70]	2020
**Total (95% CI)**			**129**			**125**	**100.0%**	**0.63 [0.38, 0.89]**	

Heterogeneity: $Tau^2 = 0.00$; $Chi^2 = 6.30$, df = 7 (P = 0.51); $I^2 = 0\%$
Test for overall effect: Z = 4.85 (P < 0.00001)

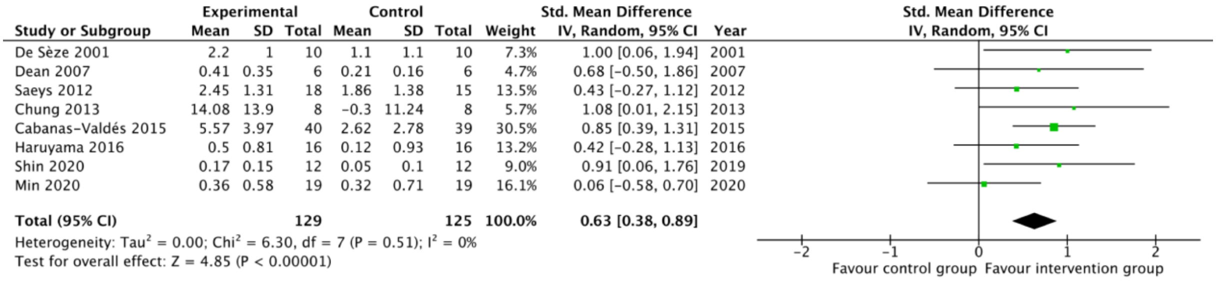

Figure S4. Pooled effect sizes on gait performance.

Study or Subgroup	Experimental Mean	Experimental SD	Experimental Total	Control Mean	Control SD	Control Total	Weight	Mean Difference IV, Random, 95% CI	Year
Chung 2013	5.42	5.61	8	5.48	6.8	8	18.0%	−0.06 [−6.17, 6.05]	2013
Jung 2014	5	11.77	9	2.6	7.52	8	11.0%	2.40 [−6.89, 11.69]	2014
Haruyama 2016	13.67	23.05	16	1.33	25.19	16	4.3%	12.34 [−4.39, 29.07]	2016
Shin 2016	9.71	4.46	12	2.18	1.13	12	29.9%	7.53 [4.93, 10.13]	2016
An 2017	4.11	2.24	15	2	3.05	14	32.0%	2.11 [0.15, 4.07]	2017
Min 2020	11.69	21.27	19	18.32	28.07	19	4.8%	−6.63 [−22.47, 9.21]	2020
**Total (95% CI)**			**79**			**77**	**100.0%**	**3.40 [−0.32, 7.12]**	

Heterogeneity: $Tau^2 = 10.25$; $Chi^2 = 15.03$, df = 5 (P = 0.01); $I^2 = 67\%$
Test for overall effect: Z = 1.79 (P = 0.07)

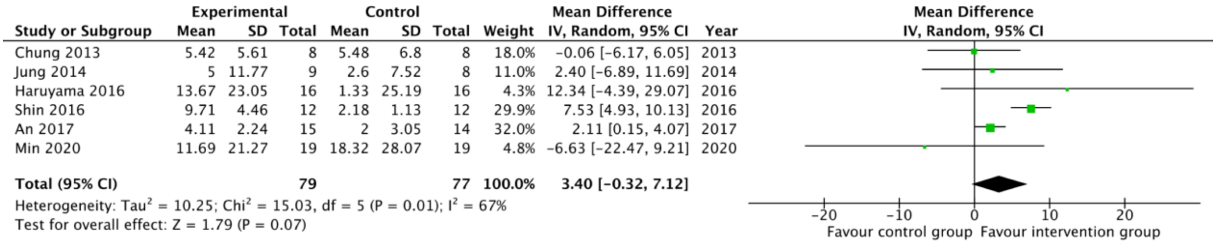

Figure S5. Pooled effect sizes on functional mobility.

Subgroup analyses for the moderator variables analyzed

A1)

Study or Subgroup	Experimental Mean	Experimental SD	Experimental Total	Control Mean	Control SD	Control Total	Weight	Std. Mean Difference IV, Random, 95% CI	Year
De Sèze 2001	33.7	11.5	10	20	18.3	10	12.6%	0.86 [−0.07, 1.78]	2001
Yoo 2010	4.78	2.69	17	2.45	4.31	31	18.1%	0.60 [−0.01, 1.20]	2010
Lee 2011	3.7	2.3	14	0.9	1.4	14	13.8%	1.43 [0.58, 2.27]	2011
Vijayakumar 2011	6.96	1.28	10	3.13	1.24	10	8.0%	2.91 [1.58, 4.25]	2011
Saeys 2012	8.72	2.8	18	2.87	3.23	15	13.9%	1.90 [1.06, 2.74]	2012
Cabanas-Valdés 2015	5.88	3.48	40	2.5	2.2	39	20.7%	1.15 [0.67, 1.62]	2015
Shin 2016	3.08	2.71	12	0.09	1.24	12	12.9%	1.37 [0.46, 2.28]	2016
**Total (95% CI)**			**121**			**131**	**100.0%**	**1.32 [0.87, 1.78]**	

Heterogeneity: $Tau^2 = 0.20$; $Chi^2 = 13.75$, df = 6 ($P = 0.03$); $I^2 = 56\%$
Test for overall effect: $Z = 5.76$ ($P < 0.00001$)

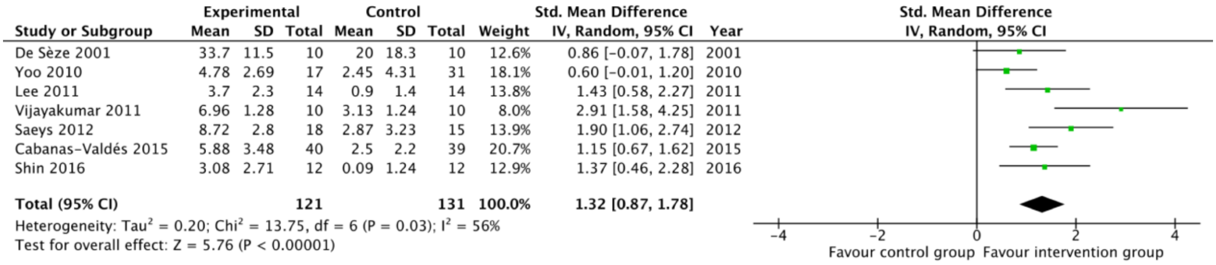

A2)

Study or Subgroup	Experimental Mean	Experimental SD	Experimental Total	Control Mean	Control SD	Control Total	Weight	Std. Mean Difference IV, Random, 95% CI	Year
Verheyden 2009	4.82	2.69	17	3.31	3.59	16	21.7%	0.47 [−0.23, 1.16]	2009
Jung 2014	2.4	1.5	9	0.1	2.38	8	10.8%	1.11 [0.07, 2.16]	2014
Jung 2015	2.36	2.94	11	−0.27	1.27	11	13.7%	1.12 [0.21, 2.03]	2015
Rose 2016	1.83	1.28	12	1.75	2.19	12	17.2%	0.04 [−0.76, 0.84]	2016
Haruyama 2016	4.13	2.38	16	1.19	2.79	16	19.1%	1.11 [0.35, 1.86]	2016
Park 2019	3.07	2.04	14	0.93	2.23	14	17.5%	0.97 [0.18, 1.76]	2018
**Total (95% CI)**			**79**			**77**	**100.0%**	**0.76 [0.40, 1.12]**	

Heterogeneity: $Tau^2 = 0.03$; $Chi^2 = 5.89$, df = 5 ($P = 0.32$); $I^2 = 15\%$
Test for overall effect: $Z = 4.15$ ($P < 0.0001$)

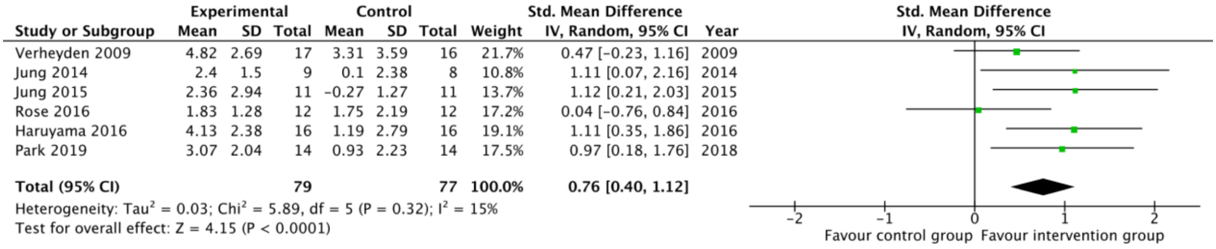

B1)

Study or Subgroup	Experimental Mean	Experimental SD	Experimental Total	Control Mean	Control SD	Control Total	Weight	Std. Mean Difference IV, Random, 95% CI	Year
De Sèze 2001	1.6	0.5	10	0.8	0.7	10	16.7%	1.26 [0.28, 2.24]	2001
Yoo 2010	11.29	13.25	28	6.32	11.99	31	24.7%	0.39 [−0.13, 0.91]	2010
Vijayakumar 2011	6.96	1.28	10	3.13	1.24	10	12.0%	2.91 [1.58, 4.25]	2011
Saeys 2012	19.39	12.61	18	9.2	13.52	15	21.2%	0.76 [0.05, 1.48]	2012
Cabanas-Valdés 2015	23.03	15.95	40	8.49	8.74	39	25.4%	1.12 [0.64, 1.59]	2015
**Total (95% CI)**			**106**			**105**	**100.0%**	**1.10 [0.51, 1.70]**	

Heterogeneity: $Tau^2 = 0.31$; $Chi^2 = 13.91$, df = 4 ($P = 0.008$); $I^2 = 71\%$
Test for overall effect: $Z = 3.62$ ($P = 0.0003$)

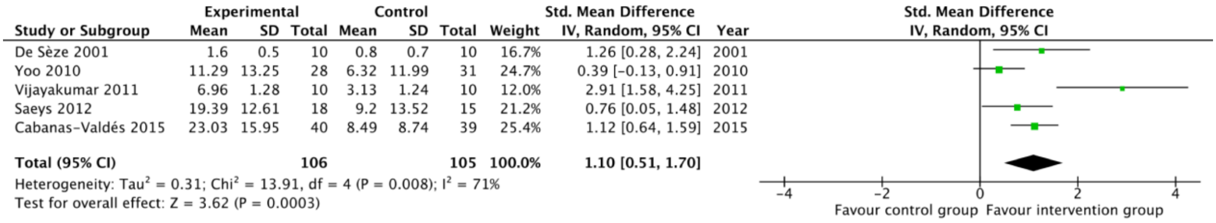

B2)

Study or Subgroup	Experimental Mean	Experimental SD	Experimental Total	Control Mean	Control SD	Control Total	Weight	Std. Mean Difference IV, Random, 95% CI	Year
Haruyama 2016	2.79	3.34	16	0.06	4.32	16	53.0%	0.69 [−0.03, 1.41]	2016
Park 2019	4.79	3.06	14	2.86	3.19	14	47.0%	0.60 [−0.16, 1.36]	2018
**Total (95% CI)**			**30**			**30**	**100.0%**	**0.65 [0.13, 1.17]**	

Heterogeneity: $Tau^2 = 0.00$; $Chi^2 = 0.03$, df = 1 ($P = 0.87$); $I^2 = 0\%$
Test for overall effect: $Z = 2.43$ ($P = 0.01$)

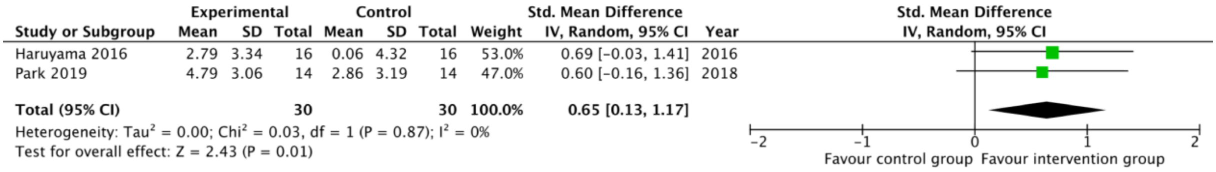

C1)

Study or Subgroup	Experimental Mean	Experimental SD	Experimental Total	Control Mean	Control SD	Control Total	Weight	Std. Mean Difference IV, Random, 95% CI	Year
Lee 2011	16.5	9.7	14	4.1	5.6	14	54.5%	1.52 [0.66, 2.38]	2011
Shin 2016	7.36	5.5	12	0.99	0.63	12	45.5%	1.57 [0.63, 2.51]	2016
**Total (95% CI)**			**26**			**26**	**100.0%**	**1.54 [0.91, 2.18]**	

Heterogeneity: $Tau^2 = 0.00$; $Chi^2 = 0.01$, df = 1 ($P = 0.94$); $I^2 = 0\%$
Test for overall effect: $Z = 4.79$ ($P < 0.00001$)

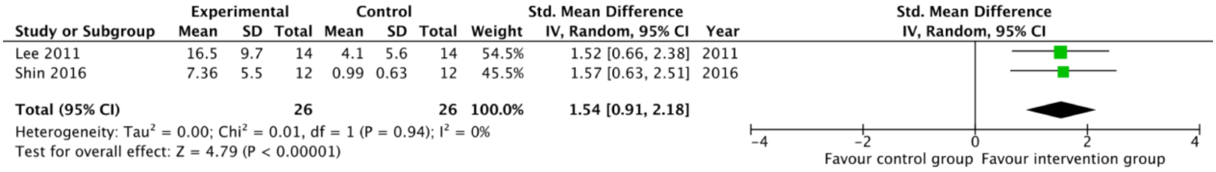

C2)

Study or Subgroup	Experimental Mean	Experimental SD	Experimental Total	Control Mean	Control SD	Control Total	Weight	Std. Mean Difference IV, Random, 95% CI	Year
Jung 2015	8.28	5.42	11	2.37	6.15	11	24.6%	0.98 [0.09, 1.88]	2015
Haruyama 2016	3.37	4.47	16	2.28	6.49	16	40.9%	0.19 [−0.50, 0.89]	2016
Park 2019	5.69	6.24	14	2.71	4.21	14	34.5%	0.54 [−0.21, 1.30]	2018
**Total (95% CI)**			**41**			**41**	**100.0%**	**0.51 [0.06, 0.95]**	

Heterogeneity: $Tau^2 = 0.00$; $Chi^2 = 1.88$, df = 2 ($P = 0.39$); $I^2 = 0\%$
Test for overall effect: $Z = 2.24$ ($P = 0.03$)

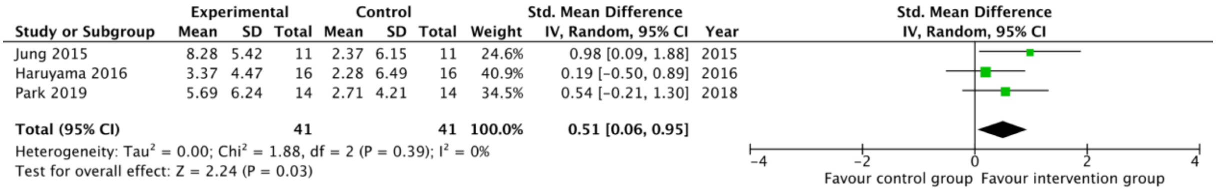

Figure S6. Subgroup analyses by initial trunk impairment. (A1) Effect on trunk function for studies below the median; (A2) Effect on trunk function for studies over the median; (B1) Effect on balance ability for studies below the median; (B2) Effect on balance ability for studies over the median; (C1) Effect on limits of stability forward reach of the unaffected arm for studies below the median; (C2) Effect on limits of stability forward reach of the unaffected arm for studies over the median.

A1)

Study or Subgroup	Experimental Mean	Experimental SD	Experimental Total	Control Mean	Control SD	Control Total	Weight	Std. Mean Difference IV, Random, 95% CI	Year
Verheyden 2009	4.82	2.69	17	3.31	3.59	16	19.6%	0.47 [−0.23, 1.16]	2009
Vijayakumar 2011	6.96	1.28	10	3.13	1.24	10	12.0%	2.91 [1.58, 4.25]	2011
Jung 2014	2.4	1.5	9	0.1	2.38	8	15.1%	1.11 [0.07, 2.16]	2014
Jung 2015	2.36	2.94	11	−0.27	1.27	11	16.7%	1.12 [0.21, 2.03]	2015
Rose 2016	1.83	1.28	12	1.75	2.19	12	18.2%	0.04 [−0.76, 0.84]	2016
Park 2019	3.07	2.04	14	0.93	2.23	14	18.3%	0.97 [0.18, 1.76]	2018
**Total (95% CI)**			**73**			**71**	**100.0%**	**0.98 [0.35, 1.61]**	

Heterogeneity: $Tau^2 = 0.40$; $Chi^2 = 14.90$, df = 5 (P = 0.01); $I^2 = 66\%$
Test for overall effect: Z = 3.05 (P = 0.002)

Std. Mean Difference IV, Random, 95% CI

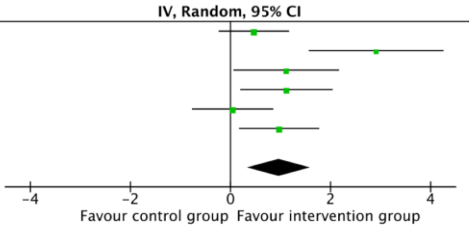

A2)

Study or Subgroup	Experimental Mean	Experimental SD	Experimental Total	Control Mean	Control SD	Control Total	Weight	Std. Mean Difference IV, Random, 95% CI	Year
De Sèze 2001	33.7	11.5	10	20	18.3	10	10.0%	0.86 [−0.07, 1.78]	2001
Yoo 2010	4.78	4.55	28	2.45	4.31	31	20.7%	0.52 [−0.00, 1.04]	2010
Lee 2011	3.7	2.3	14	0.9	1.4	14	11.5%	1.43 [0.58, 2.27]	2011
Saeys 2012	8.72	2.8	18	2.87	3.23	15	11.5%	1.90 [1.06, 2.74]	2012
Cabanas-Valdés 2015	5.88	3.48	40	2.5	2.2	39	22.4%	1.15 [0.67, 1.62]	2015
Haruyama 2016	4.13	2.38	16	1.19	2.79	16	13.5%	1.11 [0.35, 1.86]	2016
Shin 2016	3.08	2.71	12	0.09	1.24	12	10.4%	1.37 [0.46, 2.28]	2016
**Total (95% CI)**			**138**			**137**	**100.0%**	**1.13 [0.79, 1.46]**	

Heterogeneity: $Tau^2 = 0.07$; $Chi^2 = 9.46$, df = 6 (P = 0.15); $I^2 = 37\%$
Test for overall effect: Z = 6.52 (P < 0.00001)

Std. Mean Difference IV, Random, 95% CI

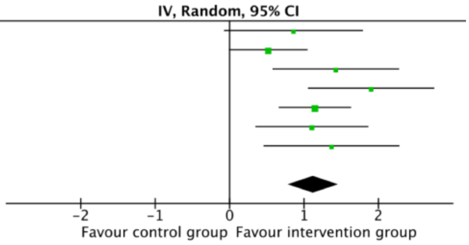

B1)

Study or Subgroup	Experimental Mean	Experimental SD	Experimental Total	Control Mean	Control SD	Control Total	Weight	Std. Mean Difference IV, Random, 95% CI	Year
Vijayakumar 2011	7.1	0.88	10	4.4	1.1	10	27.4%	2.60 [1.34, 3.85]	2011
An 2017	2.93	2.96	15	1.36	2.84	14	36.4%	0.53 [−0.22, 1.27]	2017
Park 2019	4.79	3.06	14	2.86	3.19	14	36.1%	0.60 [−0.16, 1.36]	2018
**Total (95% CI)**			**39**			**38**	**100.0%**	**1.12 [0.06, 2.18]**	

Heterogeneity: $Tau^2 = 0.66$; $Chi^2 = 8.58$, df = 2 (P = 0.01); $I^2 = 77\%$
Test for overall effect: Z = 2.06 (P = 0.04)

Std. Mean Difference IV, Random, 95% CI

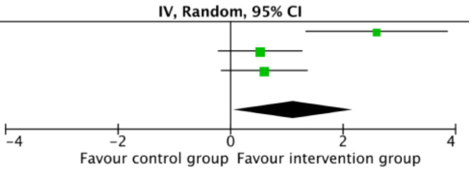

B2)

Study or Subgroup	Experimental Mean	Experimental SD	Experimental Total	Control Mean	Control SD	Control Total	Weight	Std. Mean Difference IV, Random, 95% CI	Year
De Sèze 2001	1.6	0.5	10	0.8	0.7	10	6.9%	1.26 [0.28, 2.24]	2001
Yoo 2010	11.29	13.25	28	6.32	11.99	31	24.1%	0.39 [−0.13, 0.91]	2010
Saeys 2012	19.39	12.61	18	9.2	13.52	15	12.9%	0.76 [0.05, 1.48]	2012
Cabanas-Valdés 2015	23.03	15.95	40	8.49	8.74	39	28.2%	1.12 [0.64, 1.59]	2015
Haruyama 2016	2.79	3.34	16	0.06	4.32	16	12.8%	0.69 [−0.03, 1.41]	2016
Min 2020	7.36	3.63	19	4.42	4.49	19	15.1%	0.71 [0.05, 1.36]	2020
**Total (95% CI)**			**131**			**130**	**100.0%**	**0.79 [0.53, 1.05]**	

Heterogeneity: $Tau^2 = 0.00$; $Chi^2 = 5.15$, df = 5 (P = 0.40); $I^2 = 3\%$
Test for overall effect: Z = 5.96 (P < 0.00001)

Std. Mean Difference IV, Random, 95% CI

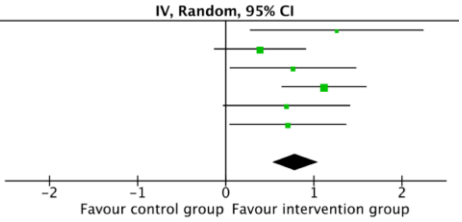

C1)

Study or Subgroup	Experimental Mean	Experimental SD	Experimental Total	Control Mean	Control SD	Control Total	Weight	Std. Mean Difference IV, Random, 95% CI	Year
Kim 2011	4.6	5.54	20	0.1	4.1	20	43.8%	0.91 [0.25, 1.56]	2011
Jung 2015	8.28	5.42	11	2.37	6.15	11	23.4%	0.98 [0.09, 1.88]	2015
Park 2019	5.69	6.24	14	2.71	4.21	14	32.8%	0.54 [−0.21, 1.30]	2018
**Total (95% CI)**			**45**			**45**	**100.0%**	**0.80 [0.37, 1.24]**	

Heterogeneity: $Tau^2 = 0.00$; $Chi^2 = 0.70$, df = 2 (P = 0.71); $I^2 = 0\%$
Test for overall effect: Z = 3.64 (P = 0.0003)

Std. Mean Difference IV, Random, 95% CI

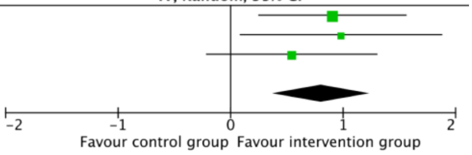

C2)

Study or Subgroup	Experimental Mean	Experimental SD	Experimental Total	Control Mean	Control SD	Control Total	Weight	Std. Mean Difference IV, Random, 95% CI	Year
Lee 2011	16.5	9.7	14	4.1	5.6	14	32.8%	1.52 [0.66, 2.38]	2011
Haruyama 2016	3.37	4.47	16	2.28	6.49	16	36.1%	0.19 [−0.50, 0.89]	2016
Shin 2016	7.36	5.5	12	0.99	0.63	12	31.2%	1.57 [0.63, 2.51]	2016
**Total (95% CI)**			**42**			**42**	**100.0%**	**1.06 [0.11, 2.01]**	

Heterogeneity: $Tau^2 = 0.53$; $Chi^2 = 7.98$, df = 2 (P = 0.02); $I^2 = 75\%$
Test for overall effect: Z = 2.18 (P = 0.03)

Std. Mean Difference IV, Random, 95% CI

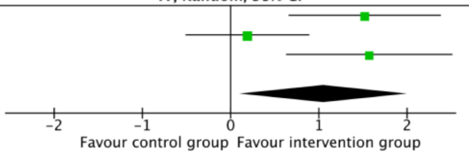

D1)

Study or Subgroup	Experimental Mean	Experimental SD	Experimental Total	Control Mean	Control SD	Control Total	Weight	Mean Difference IV, Random, 95% CI	Year
Chung 2013	5.42	5.61	8	5.48	6.8	8	9.0%	−0.06 [−6.17, 6.05]	2013
Jung 2014	5	11.77	9	2.6	7.52	8	3.9%	2.40 [−6.89, 11.69]	2014
An 2017	4.11	2.24	15	2	3.05	14	87.2%	2.11 [0.15, 4.07]	2017
**Total (95% CI)**			**32**			**30**	**100.0%**	**1.93 [0.10, 3.76]**	

Heterogeneity: $Tau^2 = 0.00$; $Chi^2 = 0.45$, df = 2 (P = 0.80); $I^2 = 0\%$
Test for overall effect: Z = 2.06 (P = 0.04)

Mean Difference IV, Random, 95% CI

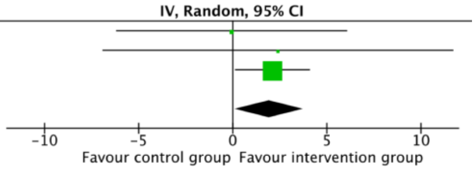

D2)

Study or Subgroup	Experimental Mean	SD	Total	Control Mean	SD	Total	Weight	Mean Difference IV, Random, 95% CI	Year
Haruyama 2016	13.67	23.05	16	1.33	25.19	16	17.2%	12.34 [−4.39, 29.07]	2016
Shin 2016	9.71	4.46	12	2.18	1.13	12	64.2%	7.53 [4.93, 10.13]	2016
Min 2020	11.69	21.27	19	18.32	28.07	19	18.6%	−6.63 [−22.47, 9.21]	2020
**Total (95% CI)**			**47**			**47**	**100.0%**	**5.72 [−2.27, 13.72]**	

Heterogeneity: $Tau^2 = 24.14$; $Chi^2 = 3.35$, df = 2 (P = 0.19); $I^2 = 40\%$
Test for overall effect: Z = 1.40 (P = 0.16)

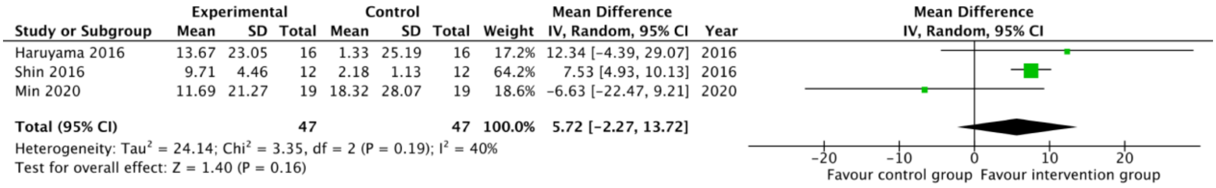

Figure S7. Subgroup analyses by participants' age. (A1) Effect on trunk function for studies below the median; (A2) Effect on trunk function for studies over the median; (B1) Effect on balance ability for studies below the median; (B2) Effect on balance ability for studies over the median (C1) Effect on limits of stability forward reach of the unaffected arm for studies below the median; (C2) Effect on limits of stability forward reach of the unaffected arm for studies over the median; (D1) Effects on functional mobility for studies below the median; (D2) Effects on functional mobility for studies over the median.

A1)

Study or Subgroup	Experimental Mean	SD	Total	Control Mean	SD	Total	Weight	Std. Mean Difference IV, Random, 95% CI	Year
De Sèze 2001	33.7	11.5	10	20	18.3	10	12.3%	0.86 [−0.07, 1.78]	2001
Verheyden 2009	4.82	2.69	17	3.31	3.59	16	15.3%	0.47 [−0.23, 1.16]	2009
Yoo 2010	4.78	4.55	28	2.45	4.31	31	17.9%	0.52 [−0.00, 1.04]	2010
Vijayakumar 2011	6.96	1.28	10	3.13	1.24	10	8.2%	2.91 [1.58, 4.25]	2011
Saeys 2012	8.72	2.8	18	2.87	3.23	15	13.3%	1.90 [1.06, 2.74]	2012
Cabanas-Valdés 2015	5.88	3.48	40	2.5	2.2	39	18.5%	1.15 [0.67, 1.62]	2015
Haruyama 2016	4.13	2.38	16	1.19	2.79	16	14.5%	1.11 [0.35, 1.86]	2016
**Total (95% CI)**			**139**			**137**	**100.0%**	**1.13 [0.65, 1.61]**	

Heterogeneity: $Tau^2 = 0.26$; $Chi^2 = 18.35$, df = 6 (P = 0.005); $I^2 = 67\%$
Test for overall effect: Z = 4.64 (P < 0.00001)

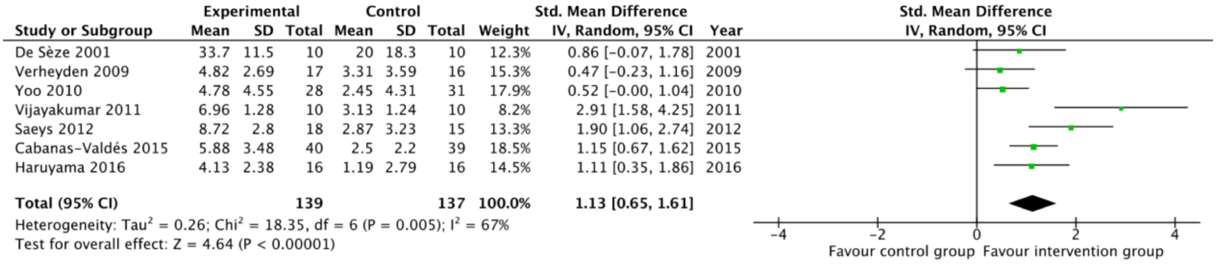

A2)

Study or Subgroup	Experimental Mean	SD	Total	Control Mean	SD	Total	Weight	Std. Mean Difference IV, Random, 95% CI	Year
Lee 2011	3.7	2.3	14	0.9	1.4	14	17.5%	1.43 [0.58, 2.27]	2011
Jung 2014	2.4	1.5	9	0.1	2.38	8	12.9%	1.11 [0.07, 2.16]	2014
Jung 2015	2.36	2.94	11	−0.27	1.27	11	15.7%	1.12 [0.21, 2.03]	2015
Shin 2016	3.08	2.71	12	0.09	1.24	12	15.9%	1.37 [0.46, 2.28]	2016
Rose 2016	1.83	1.28	12	1.75	2.19	12	18.8%	0.04 [−0.76, 0.84]	2016
Park 2019	3.07	2.04	14	0.93	2.23	14	19.1%	0.97 [0.18, 1.76]	2018
**Total (95% CI)**			**72**			**71**	**100.0%**	**0.98 [0.55, 1.41]**	

Heterogeneity: $Tau^2 = 0.09$; $Chi^2 = 7.21$, df = 5 (P = 0.21); $I^2 = 31\%$
Test for overall effect: Z = 4.49 (P < 0.00001)

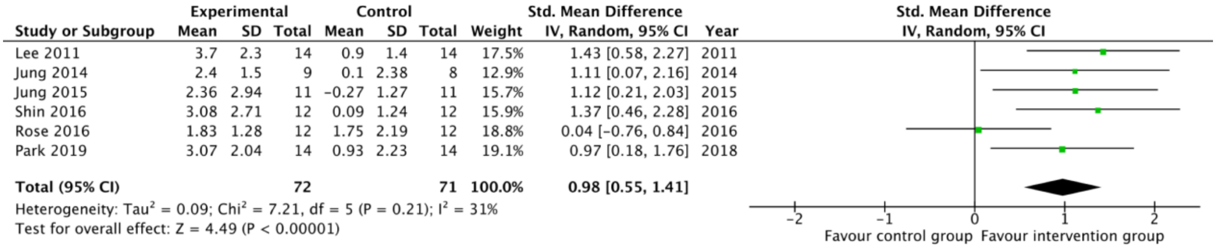

B1)

Study or Subgroup	Experimental Mean	SD	Total	Control Mean	SD	Total	Weight	Std. Mean Difference IV, Random, 95% CI	Year
De Sèze 2001	1.6	0.5	10	0.8	0.7	10	12.6%	1.26 [0.28, 2.24]	2001
Yoo 2010	11.29	13.25	28	6.32	11.99	31	21.5%	0.39 [−0.13, 0.91]	2010
Vijayakumar 2011	7.1	0.88	10	4.4	1.1	10	9.2%	2.60 [1.34, 3.85]	2011
Saeys 2012	19.39	12.61	18	9.2	13.52	15	17.2%	0.76 [0.05, 1.48]	2012
Cabanas-Valdés 2015	23.03	15.95	40	8.49	8.74	39	22.4%	1.12 [0.64, 1.59]	2015
Haruyama 2016	2.79	3.34	16	0.06	4.32	16	17.2%	0.69 [−0.03, 1.41]	2016
**Total (95% CI)**			**122**			**121**	**100.0%**	**0.98 [0.52, 1.44]**	

Heterogeneity: $Tau^2 = 0.18$; $Chi^2 = 12.55$, df = 5 (P = 0.03); $I^2 = 60\%$
Test for overall effect: Z = 4.20 (P < 0.0001)

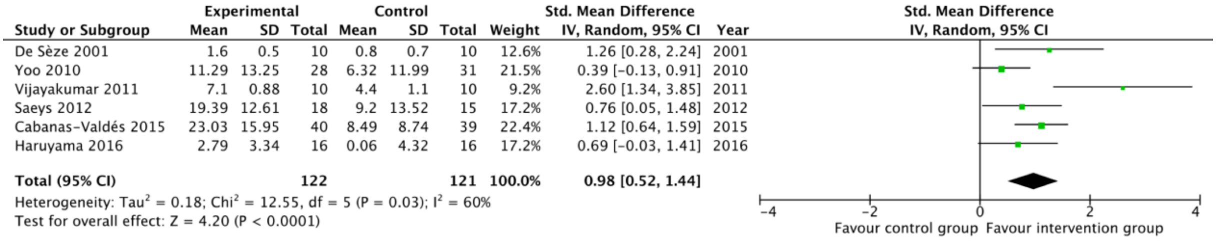

B2)

Study or Subgroup	Experimental Mean	SD	Total	Control Mean	SD	Total	Weight	Std. Mean Difference IV, Random, 95% CI	Year
An 2017	2.93	2.96	15	1.36	2.84	14	30.9%	0.53 [−0.22, 1.27]	2017
Park 2019	4.79	3.06	14	2.86	3.19	14	29.6%	0.60 [−0.16, 1.36]	2018
Min 2020	7.36	3.63	19	4.42	4.49	19	39.5%	0.71 [0.05, 1.36]	2020
**Total (95% CI)**			**48**			**47**	**100.0%**	**0.62 [0.21, 1.03]**	

Heterogeneity: $Tau^2 = 0.00$; $Chi^2 = 0.13$, df = 2 (P = 0.94); $I^2 = 0\%$
Test for overall effect: Z = 2.93 (P = 0.003)

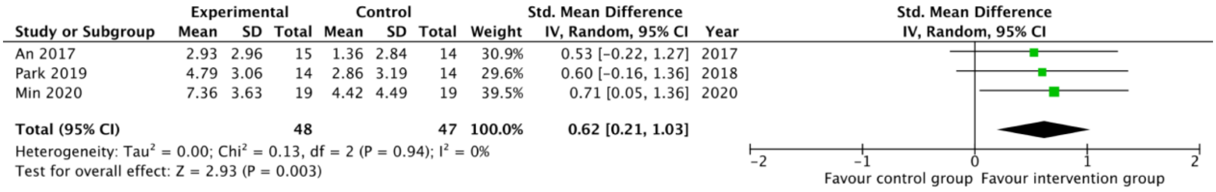

C1)

Study or Subgroup	Experimental Mean	SD	Total	Control Mean	SD	Total	Weight	Std. Mean Difference IV, Random, 95% CI	Year
De Sèze 2001	2.2	1	10	1.1	1.1	10	13.1%	1.00 [0.06, 1.94]	2001
Dean 2007	0.41	0.35	6	0.21	0.16	6	8.4%	0.68 [−0.50, 1.86]	2007
Cabanas-Valdés 2015	5.57	3.97	40	2.62	2.78	39	54.8%	0.85 [0.39, 1.31]	2015
Haruyama 2016	0.5	0.81	16	0.12	0.93	16	23.7%	0.42 [−0.28, 1.13]	2016
**Total (95% CI)**			**72**			**71**	**100.0%**	**0.76 [0.41, 1.10]**	

Heterogeneity: $Tau^2 = 0.00$; $Chi^2 = 1.30$, df = 3 (P = 0.73); $I^2 = 0\%$
Test for overall effect: Z = 4.33 (P < 0.0001)

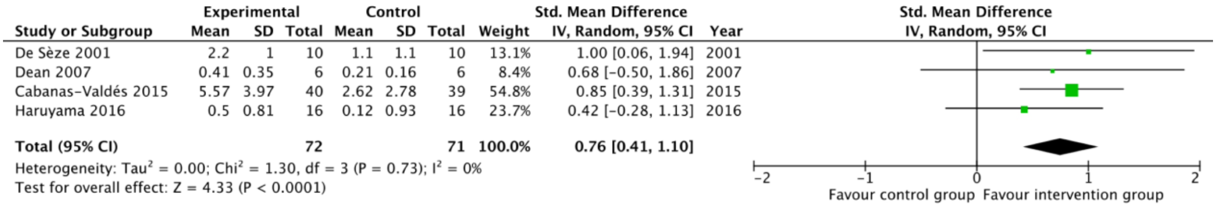

C2)

Study or Subgroup	Experimental Mean	Experimental SD	Experimental Total	Control Mean	Control SD	Control Total	Weight	Std. Mean Difference IV, Random, 95% CI	Year
Chung 2013	14.08	13.9	8	−0.3	11.24	8	24.9%	1.08 [0.01, 2.15]	2013
Shin 2020	0.17	0.15	12	0.05	0.1	12	32.6%	0.91 [0.06, 1.76]	2019
Min 2020	0.36	0.58	19	0.32	0.71	19	42.5%	0.06 [−0.58, 0.70]	2020
**Total (95% CI)**			**39**			**39**	**100.0%**	**0.59 [−0.08, 1.26]**	

Heterogeneity: $Tau^2 = 0.17$; $Chi^2 = 3.84$, df = 2 (P = 0.15); $I^2 = 48\%$
Test for overall effect: Z = 1.74 (P = 0.08)

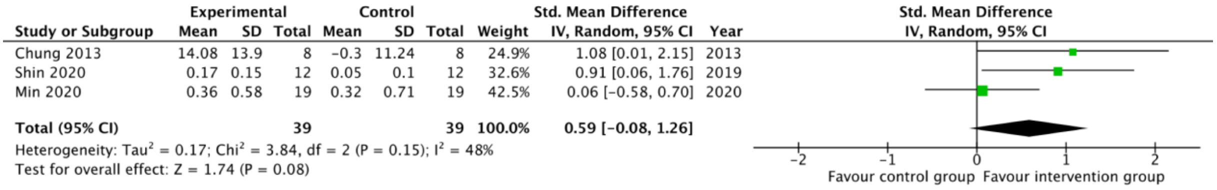

Figure S8. Subgroup analyses by the start of the intervention after the stroke-onset. (A1) Effect on trunk function for studies below the median; (A2) Effect on trunk function for studies over the median; (B1) Effect on balance ability for studies below the median; (B2) Effect on balance ability for studies over the median; (C1) Effect on gait performance for studies below the median; (C2) Effect on gait performance for studies over the median.

A1)

Study or Subgroup	Experimental Mean	Experimental SD	Experimental Total	Control Mean	Control SD	Control Total	Weight	Std. Mean Difference IV, Random, 95% CI	Year
Yoo 2010	4.78	4.55	28	2.45	4.31	31	33.4%	0.52 [−0.00, 1.04]	2010
Cabanas-Valdés 2015	5.88	3.48	40	2.5	2.2	39	36.9%	1.15 [0.67, 1.62]	2015
Jung 2015	2.36	2.94	11	−0.27	1.27	11	14.8%	1.12 [0.21, 2.03]	2015
Shin 2016	3.08	2.71	12	0.09	1.24	12	14.9%	1.37 [0.46, 2.28]	2016
**Total (95% CI)**			**91**			**93**	**100.0%**	**0.97 [0.58, 1.35]**	

Heterogeneity: $Tau^2 = 0.05$; $Chi^2 = 4.24$, df = 3 (P = 0.24); $I^2 = 29\%$
Test for overall effect: Z = 4.91 (P < 0.00001)

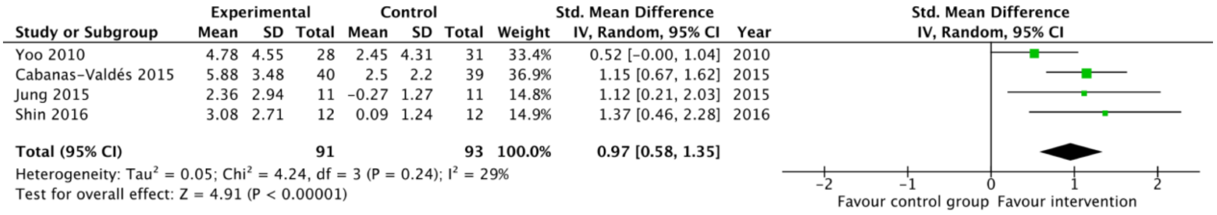

A2)

Study or Subgroup	Experimental Mean	Experimental SD	Experimental Total	Control Mean	Control SD	Control Total	Weight	Std. Mean Difference IV, Random, 95% CI	Year
De Sèze 2001	33.7	11.5	10	20	18.3	10	11.9%	0.86 [−0.07, 1.78]	2001
Verheyden 2009	4.82	2.69	17	3.31	3.59	16	15.4%	0.47 [−0.23, 1.16]	2009
Vijayakumar 2011	6.96	1.28	10	3.13	1.24	10	7.6%	2.91 [1.58, 4.25]	2011
Lee 2011	3.7	2.3	14	0.9	1.4	14	13.1%	1.43 [0.58, 2.27]	2011
Saeys 2012	8.72	2.8	18	2.87	3.23	15	13.1%	1.90 [1.06, 2.74]	2012
Jung 2014	2.4	1.5	9	0.1	2.38	8	10.4%	1.11 [0.07, 2.16]	2014
Haruyama 2016	4.13	2.38	16	1.19	2.79	16	14.5%	1.11 [0.35, 1.86]	2016
Park 2019	3.07	2.04	14	0.93	2.23	14	13.9%	0.97 [0.18, 1.76]	2018
**Total (95% CI)**			**108**			**103**	**100.0%**	**1.24 [0.80, 1.69]**	

Heterogeneity: $Tau^2 = 0.21$; $Chi^2 = 14.46$, df = 7 (P = 0.04); $I^2 = 52\%$
Test for overall effect: Z = 5.50 (P < 0.00001)

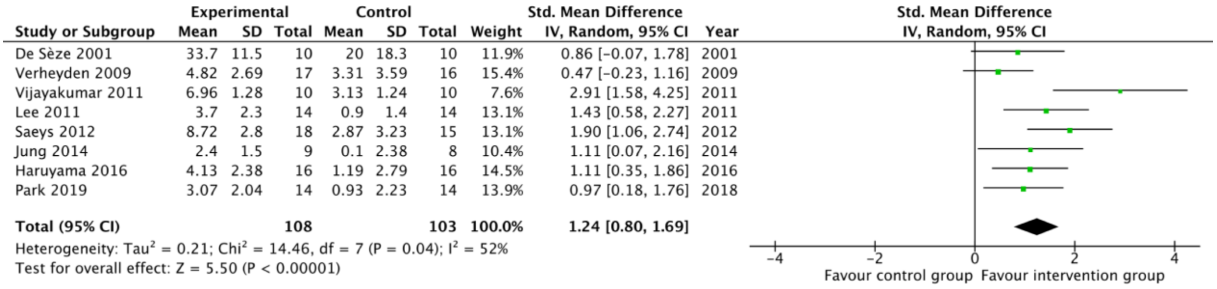

B1)

Study or Subgroup	Experimental Mean	Experimental SD	Experimental Total	Control Mean	Control SD	Control Total	Weight	Std. Mean Difference IV, Random, 95% CI	Year
Yoo 2010	11.29	13.25	28	6.32	11.99	31	36.2%	0.39 [−0.13, 0.91]	2010
Cabanas-Valdés 2015	23.03	15.95	40	8.49	8.74	39	38.5%	1.12 [0.64, 1.59]	2015
An 2017	2.93	2.96	15	1.36	2.84	14	25.3%	0.53 [−0.22, 1.27]	2017
**Total (95% CI)**			**83**			**84**	**100.0%**	**0.70 [0.21, 1.19]**	

Heterogeneity: $Tau^2 = 0.10$; $Chi^2 = 4.49$, df = 2 (P = 0.11); $I^2 = 55\%$
Test for overall effect: Z = 2.82 (P = 0.005)

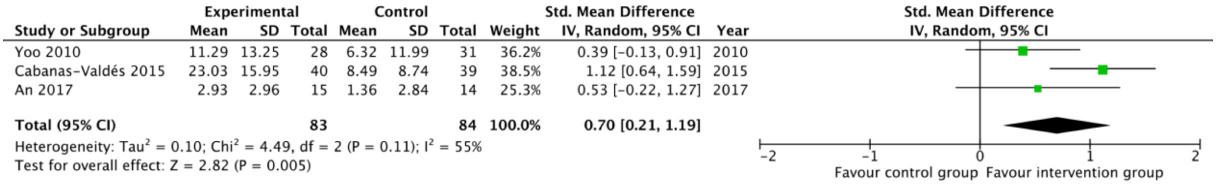

B2)

Study or Subgroup	Experimental Mean	Experimental SD	Experimental Total	Control Mean	Control SD	Control Total	Weight	Std. Mean Difference IV, Random, 95% CI	Year
De Sèze 2001	1.6	0.5	10	0.8	0.7	10	13.3%	1.26 [0.28, 2.24]	2001
Vijayakumar 2011	7.1	0.88	10	4.4	1.1	10	9.4%	2.60 [1.34, 3.85]	2011
Saeys 2012	19.39	12.61	18	9.2	13.52	15	19.3%	0.76 [0.05, 1.48]	2012
Haruyama 2016	2.79	3.34	16	0.06	4.32	16	19.2%	0.69 [−0.03, 1.41]	2016
Park 2019	4.79	3.06	14	2.86	3.19	14	18.0%	0.60 [−0.16, 1.36]	2018
Min 2020	7.36	3.63	19	4.42	4.49	19	20.8%	0.71 [0.05, 1.36]	2020
**Total (95% CI)**			**87**			**84**	**100.0%**	**0.95 [0.50, 1.39]**	

Heterogeneity: $Tau^2 = 0.13$; $Chi^2 = 8.94$, df = 5 (P = 0.11); $I^2 = 44\%$
Test for overall effect: Z = 4.20 (P < 0.0001)

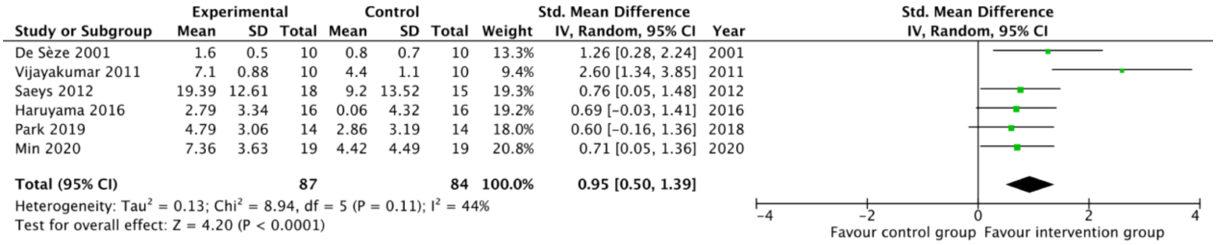

C1)

Study or Subgroup	Experimental Mean	Experimental SD	Experimental Total	Control Mean	Control SD	Control Total	Weight	Std. Mean Difference IV, Random, 95% CI	Year
Kim 2011	4.6	5.54	20	0.1	4.1	20	49.5%	0.91 [0.25, 1.56]	2011
Jung 2015	8.28	5.42	11	2.37	6.15	11	26.4%	0.98 [0.09, 1.88]	2015
Shin 2016	7.36	5.5	12	0.99	0.63	12	24.1%	1.57 [0.63, 2.51]	2016
**Total (95% CI)**			**43**			**43**	**100.0%**	**1.09 [0.63, 1.55]**	

Heterogeneity: $Tau^2 = 0.00$; $Chi^2 = 1.38$, df = 2 (P = 0.50); $I^2 = 0\%$
Test for overall effect: Z = 4.63 (P < 0.00001)

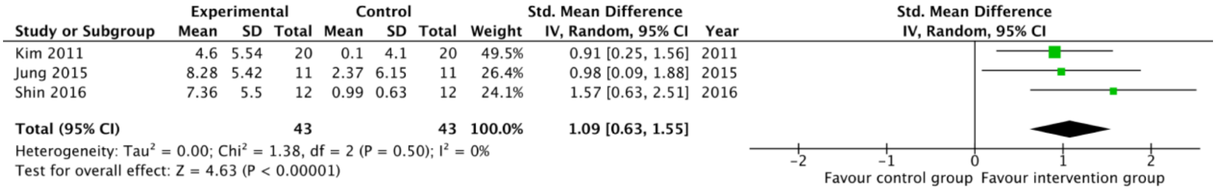

C2)

Study or Subgroup	Experimental Mean	Experimental SD	Experimental Total	Control Mean	Control SD	Control Total	Weight	Std. Mean Difference IV, Random, 95% CI	Year
Lee 2011	16.5	9.7	14	4.1	5.6	14	30.7%	1.52 [0.66, 2.38]	2011
Haruyama 2016	3.37	4.47	16	2.28	6.49	16	35.6%	0.19 [−0.50, 0.89]	2016
Park 2019	5.69	6.24	14	2.71	4.21	14	33.7%	0.54 [−0.21, 1.30]	2018
**Total (95% CI)**			**44**			**44**	**100.0%**	**0.72 [−0.03, 1.47]**	

Heterogeneity: $Tau^2 = 0.28$; $Chi^2 = 5.72$, df = 2 (P = 0.06); $I^2 = 65\%$
Test for overall effect: Z = 1.88 (P = 0.06)

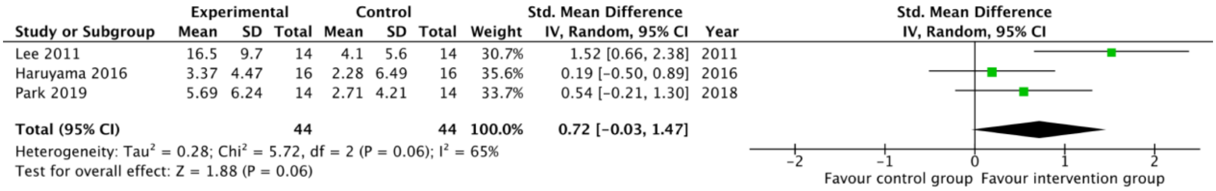

D1)

Study or Subgroup	Experimental Mean	Experimental SD	Experimental Total	Control Mean	Control SD	Control Total	Weight	Std. Mean Difference IV, Random, 95% CI	Year
Dean 2007	0.41	0.35	6	0.21	0.16	6	9.4%	0.68 [−0.50, 1.86]	2007
Chung 2013	14.08	13.9	8	−0.3	11.24	8	11.4%	1.08 [0.01, 2.15]	2013
Cabanas-Valdés 2015	5.57	3.97	40	2.62	2.78	39	61.1%	0.85 [0.39, 1.31]	2015
Shin 2020	0.17	0.15	12	0.05	0.1	12	18.1%	0.91 [0.06, 1.76]	2019
**Total (95% CI)**			**66**			**65**	**100.0%**	**0.87 [0.51, 1.23]**	

Heterogeneity: $Tau^2 = 0.00$; $Chi^2 = 0.26$, df = 3 (P = 0.97); $I^2 = 0\%$
Test for overall effect: Z = 4.73 (P < 0.00001)

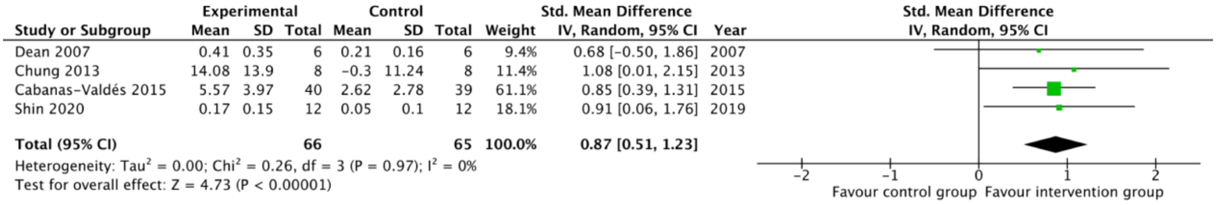

D2)

Study or Subgroup	Experimental Mean	Experimental SD	Experimental Total	Control Mean	Control SD	Control Total	Weight	Std. Mean Difference IV, Random, 95% CI	Year
De Sèze 2001	2.2	1	10	1.1	1.1	10	14.6%	1.00 [0.06, 1.94]	2001
Saeys 2012	2.45	1.31	18	1.86	1.38	15	26.9%	0.43 [−0.27, 1.12]	2012
Haruyama 2016	0.5	0.81	16	0.12	0.93	16	26.4%	0.42 [−0.28, 1.13]	2016
Min 2020	0.36	0.58	19	0.32	0.71	19	32.1%	0.06 [−0.58, 0.70]	2020
**Total (95% CI)**			**63**			**60**	**100.0%**	**0.39 [0.03, 0.75]**	

Heterogeneity: $Tau^2 = 0.00$; $Chi^2 = 2.67$, df = 3 (P = 0.44); $I^2 = 0\%$
Test for overall effect: Z = 2.14 (P = 0.03)

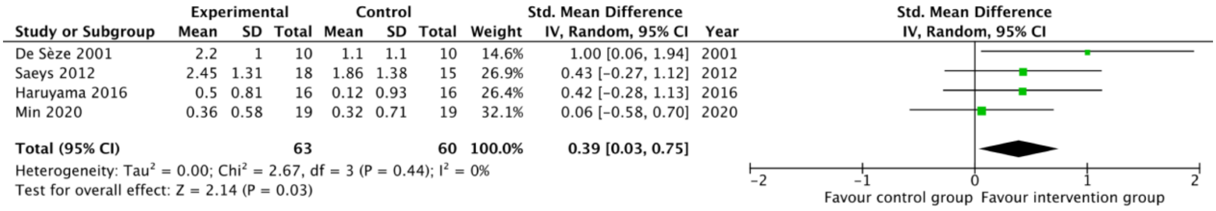

E1)

Study or Subgroup	Experimental Mean	Experimental SD	Experimental Total	Control Mean	Control SD	Control Total	Weight	Mean Difference IV, Random, 95% CI	Year
Chung 2013	5.42	5.61	8	5.48	6.8	8	23.4%	−0.06 [−6.17, 6.05]	2013
Shin 2016	9.71	4.46	12	2.18	1.13	12	37.2%	7.53 [4.93, 10.13]	2016
An 2017	4.11	2.24	15	2	3.05	14	39.4%	2.11 [0.15, 4.07]	2017
**Total (95% CI)**			**35**			**34**	**100.0%**	**3.62 [−0.78, 8.01]**	

Heterogeneity: $Tau^2 = 11.78$; $Chi^2 = 12.28$, df = 2 (P = 0.002); $I^2 = 84\%$
Test for overall effect: Z = 1.61 (P = 0.11)

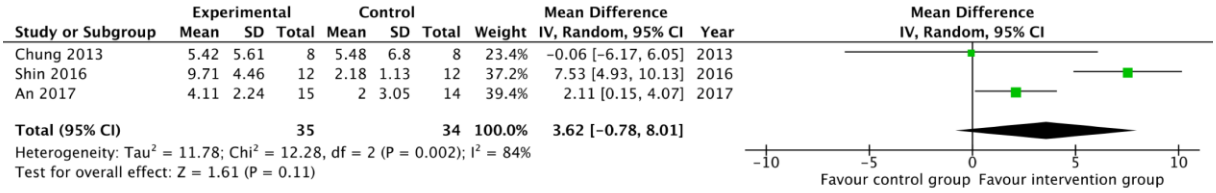

E2)

Study or Subgroup	Experimental Mean	Experimental SD	Experimental Total	Control Mean	Control SD	Control Total	Weight	Mean Difference IV, Random, 95% CI	Year
Jung 2014	5	11.77	9	2.6	7.52	8	52.9%	2.40 [−6.89, 11.69]	2014
Haruyama 2016	13.67	23.05	16	1.33	25.19	16	22.5%	12.34 [−4.39, 29.07]	2016
Min 2020	11.69	21.27	19	18.32	28.07	19	24.6%	−6.63 [−22.47, 9.21]	2020
**Total (95% CI)**			**44**			**43**	**100.0%**	**2.41 [−6.29, 11.11]**	

Heterogeneity: $Tau^2 = 14.80$; $Chi^2 = 2.61$, df = 2 (P = 0.27); $I^2 = 23\%$
Test for overall effect: Z = 0.54 (P = 0.59)

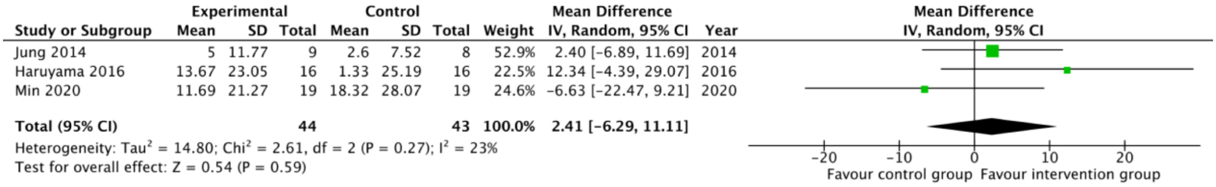

Figure S9. Subgroup analyses by total volume (minutes) of the additional trunk exercise programs. (A1) Effect on trunk function for studies below the median; (A2) Effect on trunk function for studies over the median; (B1) Effect on balance ability for studies below the median; (B2) Effect on balance ability for studies over the median; (C1) Effect on limits of stability forward reach of the unaffected arm for studies below the median; (C2) Effect on limits of stability forward reach of the unaffected arm for studies over the median; (D1) Effects on gait performance for studies below the median; (D2) Effects on gait performance for studies over the median; (E1) Effects on functional mobility for studies below the median; (E2) Effects on functional mobility for studies over the median.

Table S3. PEDro scale to assess methodological quality.

	Eligibility criteria specified	Subjects random allocation	Concealed allocation	Similar groups baseline	Subjects blinding	Therapists blinding	Assessors blinding	Outcome measurement in 85% of the subjects initially allocated	Intention to treat	Between-group statistical comparison	Point measures and variability
DeSèze et al., 2001	✔	✔	✔	✔	✗	✗	✔	✔	✔	✔	✔
Howe et al., 2005	✔	✔	✔	✔	✗	✗	✔	✔	✗	✔	✗
Dean et al., 2007	✔	✔	✔	✔	✗	✗	✔	✔	✔	✔	✔
Verheyden et al., 2009	✔	✔	✔	✔	✗	✗	✔	✔	✔	✔	✔
Yoo et al., 2010	✔	✔	✗	✔	✗	✗	✗	?	?	✔	✗
Kim et al., 2011	✔	✔	✗	✔	✗	✗	✗	✔	✔	✔	✗
Vijayakumar et al., 2011	✔	✔	✔	✔	✗	✗	✔	?	?	✔	✗
Lee et al., 2011	✔	✔	✗	✔	✗	✗	✔	✔	✗	✔	✔
Saeys et al., 2012	✔	✔	✔	✔	✗	✗	✔	✔	✔	✔	✔
Chung et al., 2013	✔	✔	✗	✔	✗	✗	✗	✔	✔	✔	✔
Jung et al., 2014	✔	✔	✔	✔	✗	✗	✔	✔	✗	✔	✗

Cabanas-Valdés et al., 2015	✔	✔	✔	✔	✗	✗	✔	✔	✗	✔	✔
Jung et al., 2015	✔	✔	✔	✔	✗	✗	✔	✗	✗	✔	✔
Haruyama et al., 2016	✔	✔	✔	✔	✗	✗	✔	✔	✗	✔	✔
Shin et al., 2016	✔	✔	✔	✔	✗	✗	✔	✔	✔	✔	✔
Rose et al., 2016	✔	✔	✔	✔	✗	✗	✔	✔	✗	✔	✔
An et al., 2017	✔	✔	✔	✔	✗	✗	?	✔	✗	✔	✔
Park et al., 2019	✔	✔	✔	✔	✔	✗	✗	✔	✗	✔	✗
Min et al., 2020	✔	✔	✔	✔	✗	✗	✔	✔	✔	✔	✔

Table S4. Quality of evidence (GRADE approach) between additional trunk-focused exercises vs conventional rehabilitation.

№ of studies	*Study design*	*Risk of bias (PEDro)*	*Inconsistency*	*Indirectness*	*Imprecision*	*Publication bias*	*Sample Experimental group*	*Sample Control group*	*Pooled effect size (95% CI)*	*Certainty*	*Importance*
					Trunk function						
13	randomised trials	serious [a]	serious [b]	serious [c]	not serious	none	211	208	SMD **1.06 SD higher** (0.74 higher to 1.37 higher)	⊕◯◯◯ VERY LOW	CRITICAL
					Balance ability						
9	randomised trials	serious [a]	not serious	serious [c]	not serious	none	170	168	SMD **0.83 SD higher** (0.52 higher to 1.14 higher)	⊕⊕◯◯ LOW	CRITICAL
					Limits of stability - Forward unaffected						
6	randomised trials	serious [a]	not serious	serious [c]	serious [d]	none	87	87	SMD **0.9 SD higher** (0.47 higher to 1.33 higher)	⊕◯◯◯ VERY LOW	CRITICAL
					Limits of stability - Lateral unaffected						
4	randomised trials	serious [a]	not serious	serious [c]	serious [d]	none	52	55	SMD **1.16 SD higher** (0.67 higher to 1.66 higher)	⊕◯◯◯ VERY LOW	CRITICAL
					Limits of stability - Lateral affected						
3	randomised trials	serious [a]	not serious	not serious	serious [d]	none	37	37	SMD **0.89 SD higher** (0.26 higher to 1.52 higher)	⊕⊕◯◯ LOW	CRITICAL
					Gait performance						
8	randomised trials	not serious	not serious	serious [c]	serious [d]	none	129	125	SMD **0.63 SD higher** (0.38 higher to 0.89 higher)	⊕⊕◯◯ LOW	CRITICAL
					Functional mobility						

6	randomised trials	serious [a]	serious [b]	not serious	very serious [d, e]	none	79	77	MD **3.4 higher** (-0.32 lower to 7.12 higher)	⊕◯◯◯ VERY LOW	CRITICAL

Abbreviations. PEDro: Physiotherapy Evidence Database Scale; CI: Confidence interval; SMD: Standardized mean difference; MD: Weighted Mean difference; I^2: Inconsistency Statistic;

a. Downgraded one level since at least two studies scored ≤6 on the PEDro scale
b. Downgraded one level due to an Inconsistency statistic (I^2) ≥ 50%
c. Downgraded one level because different test/scales were employed to measure the outcome
d. Downgraded one level due to a sample with less than 300 participants
e. Downgraded one level due to large confidence intervals (Includes the 0-Hypothesis)
